# Myofibrillar myopathy hallmarks associated with ZAK deficiency

**DOI:** 10.1093/hmg/ddad113

**Published:** 2023-07-10

**Authors:** Amy Stonadge, Aitana V Genzor, Alex Russell, Mohamed F Hamed, Norma Romero, Gareth Evans, Mary Elizabeth Pownall, Simon Bekker-Jensen, Gonzalo Blanco

**Affiliations:** York Biomedical Research Institute, Department of Biology, University of York, York, YO10 5DD, UK; Center for Healthy Aging, Department of Cellular and Molecular Medicine, University of Copenhagen, DK-2200 Copenhagen, Denmark; York Biomedical Research Institute, Department of Biology, University of York, York, YO10 5DD, UK; Department of Pathology, Faculty of Veterinary Medicine, Mansoura University, Mansoura, Egypt; Unité de Morphologie Neuromusculaire Institut de Myologie - Inserm Sorbonne Université - GHU Pitié-Salpêtrière 47- 83, boulevard de l’Hôpital F-75 651 Paris, Cedex 13, France; York Biomedical Research Institute, Department of Biology, University of York, York, YO10 5DD, UK; York Biomedical Research Institute, Department of Biology, University of York, York, YO10 5DD, UK; Center for Healthy Aging, Department of Cellular and Molecular Medicine, University of Copenhagen, DK-2200 Copenhagen, Denmark; York Biomedical Research Institute, Department of Biology, University of York, York, YO10 5DD, UK

## Abstract

The *ZAK* gene encodes two functionally distinct kinases, ZAKα and ZAKβ. Homozygous loss of function mutations affecting both isoforms causes a congenital muscle disease. ZAKβ is the only isoform expressed in skeletal muscle and is activated by muscle contraction and cellular compression. The ZAKβ substrates in skeletal muscle or the mechanism whereby ZAKβ senses mechanical stress remains to be determined. To gain insights into the pathogenic mechanism, we exploited ZAK-deficient cell lines, zebrafish, mice and a human biopsy. ZAK-deficient mice and zebrafish show a mild phenotype. In mice, comparative histopathology data from regeneration, overloading, ageing and sex conditions indicate that while age and activity are drivers of the pathology, ZAKβ appears to have a marginal role in myoblast fusion *in vitro* or muscle regeneration *in vivo.* The presence of SYNPO2, BAG3 and Filamin C (FLNC) in a phosphoproteomics assay and extended analyses suggested a role for ZAKβ in the turnover of FLNC. Immunofluorescence analysis of muscle sections from mice and a human biopsy showed evidence of FLNC and BAG3 accumulations as well as other myofibrillar myopathy markers. Moreover, endogenous overloading of skeletal muscle exacerbated the presence of fibres with FLNC accumulations in mice*,* indicating that ZAKβ signalling is necessary for an adaptive turnover of FLNC that allows for the normal physiological response to sustained mechanical stress. We suggest that accumulation of mislocalized FLNC and BAG3 in highly immunoreactive fibres contributes to the pathogenic mechanism of ZAK deficiency.

## Introduction

*ZAK* (leucine zipper and sterile alpha motif-containing kinase, also known as *MAP 3 K20*, *MRK* or *MLTK*) is a *MAP 3* kinase independently identified by four groups in the 2000s (1–4). Since then, diverse roles have been assigned to the two isoforms encoded by the *ZAK* gene, *ZAKα* and *ZAKβ*. A potential role for ZAK in muscle function was initially suggested by its association with the Kyphoscoliosis peptidase (KY) protein complex (5). The *Ky* gene was first identified in the mouse following identification of a muscular dystrophy-causing mutation (6). Subsequent protein interaction analyses revealed a putative Z-disc protein network that includes KY, ZAK, IGFN1 (immunoglobulin like and fibronectin type III domain containing 1) and others (5,7). The key roles for KY and ZAK in muscle function are now evident from identification of disruptive mutations in both genes causing congenital myopathy in rare human pedigrees (8–11). In the case of *ZAK*, two homozygous frameshift mutations and a homozygous nonsense mutation were identified in three consanguineous families from different ethnic backgrounds (11). These mutations were the first example of a direct association between a MAP 3 K and skeletal muscle weakness. In all affected individuals, fibre size variation, predominance of type I fibres and centralized nuclei are common pathological findings (11). Other evidence points at ZAK as having a key physiological role in skeletal muscle. Phosphoproteomics analysis following a bout of maximal intensity contractions showed *ZAK* as one the most abundant phosphorylated proteins (12). In mice, ZAK deficiency causes pathology in skeletal muscle only (13). The *ZAK* mutations described in mice and humans cause loss of function of both isoforms, but the pathological observations can be attributed specifically to *ZAKβ*, as this is the only isoform expressed in skeletal muscle (11).

ZAKα and ZAKβ hold a common N-terminal serine/threonine kinase domain but distinctive C-terminal domains. The C-terminal domains have been shown to confer different functions to their respective isoforms. Thus, ZAKα performs a function in ribotoxic stress sensing through two ribosome-binding domains in its C-terminus that serve as a molecular sensing module (14), whereas ZAKβ has been shown to respond to mechanical perturbation of cytoskeletal stress fibres in U2OS cells (13). Under osmotic shock or cellular compression conditions, ZAKβ auto-phosphorylates, relocates to stress fibres through its unique C-terminal domain and leads to p38 activation (13). *In vivo*, repeated contractions of *tibialis anterior* (TA) and *extensor digitorum longus* muscles in mice leads to acute ZAKβ-dependent p38 activation (13). In humans, resistance exercise specifically has been shown to strongly activate ZAKβ (15). Therefore, ZAKβ appears to play a role in the early response to mechanical stress in skeletal muscle. The mechanisms by which ZAKβ senses mechanical stress as well as the downstream panoply of ZAKβ targets remain to be determined.

Here, we first expanded the muscle histopathology data of *Zak^−/−^* mice to include challenges such as regeneration, overloading and ageing. To identify direct targets of ZAKβ, we performed a phosphoproteomics assay. Overrepresented phosphopeptides identified components of cell adhesion. Amongst these, the actin filament cross-linking protein Filamin C (FLNC) emerged as an interesting pathogenic marker. We identify hallmarks of impaired FLNC turnover and other myofibrillar myopathy markers in muscle sections from *Zak^−/−^* mice and the biopsy of a ZAK-deficient patient.

## Results

### Pathology severity is gender-biassed and increases with age

**Figure 1:**
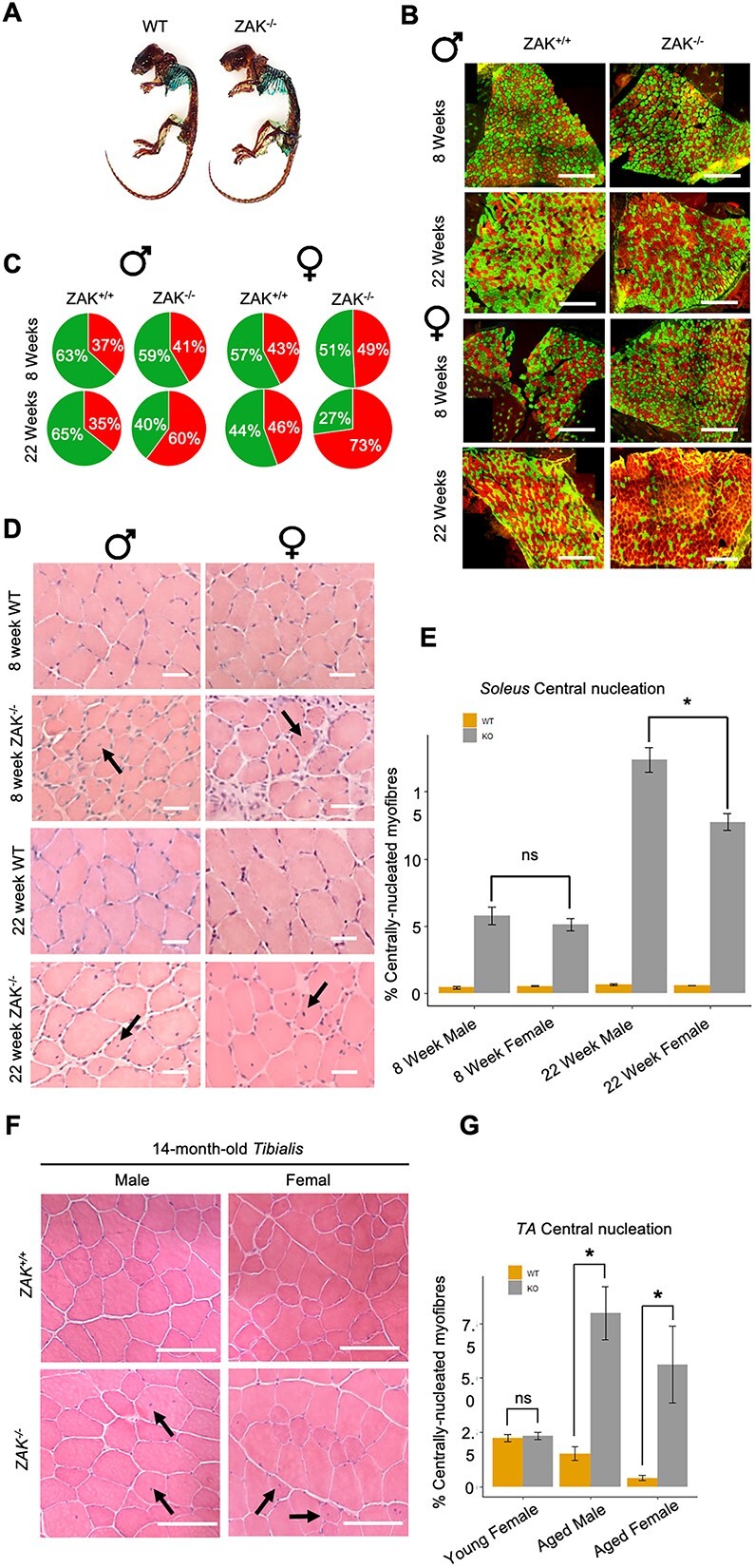
Muscle pathological changes are gender biassed in *Zak^−/−^* mice. (**A**) A whole-mount skeletal preparation from WT (left panel) and *Zak^−/−^* (right panel) mice at 24 weeks. Alizarin red (red) and Alcian blue (blue) label mineralized bone and cartilage, respectively. There is no evidence of spinal deformity in the *Zak*^−/−^ mouse. (**B**) Representative immunofluorescence of cross section of mouse *soleus* muscle stained for type I (red) and type IIa (green) fibres using anti-myosin isoform-specific antibodies in *soleus* muscle. Scale bars = 500 μm. Categories of age, sex and genotype as annotated. (**C**) Pie charts depicting the relative counts of type I (red) fibres in relation to type IIa (green) fibres in whole *soleus* muscle. Twenty-two-week *Zak^−/−^* muscles show a predominance of type I fibres that increases with age (*n* = 3). Twenty-two-week *Zak^−/−^* male versus 22-week *Zak^−/−^* female, *P* < 0.01 in ANOVA with Bonferroni–Dunn correction for multiple comparisons. Percentage of type I/IIa fibres displayed in each segment. (**D**) H&E staining of 8- and 22-week *soleus* muscle sections from *Zak^+/+^* and *Zak^−/−^* male and female mice. Black arrows point at examples of centralized nuclei used as a proxy for regenerating myofibres. Scale bars = 50 μm. (**E**) A histogram showing quantifications of (D). Values indicate the percentage of total *soleus* muscle fibres with centralized nuclei in *Zak^−/−^* and control mice at 8 and 22 weeks (*n* = 3). Twenty-two-week *Zak^−/−^* male versus 22-week *Zak^−/−^* female, ^*^P < 0.01 in ANOVA with Bonferroni–Dunn correction for multiple comparisons. (**F**) H&E staining of TA muscle cross sections from 14-month-old male and female *ZAK^−/−^* and control mice. Black arrows point at examples of internal nuclei. Scale bars = 100 μm. (**G**) A histogram showing quantifications of (F). Values indicate the percentage of total *tibialis* muscle fibres with centralized nuclei. Note increased levels of regeneration in the *TA* of male and female *Zak^−/−^* mice at 14-months-old (grey) (*n* = 6). ^*^P < 0.01 using *t*-test.

Scoliosis and developmental delays are features of ZAK-deficient patients (11). To test this in mice, we looked at skeletal muscle preparations from adult mice. The distribution of bone and cartilage did not reveal any difference in *Zak^−/−^* mice compared with control (Fig. 1A), indicating that scoliosis or any other skeletal anomalies are not a feature of ZAK deficiency in mice. To check for any gender bias, which has been occasionally reported in muscular dystrophy (16,17), we extended the morphological analysis of slow and fast muscle sections previously done (13) to include males and females (Fig. 1B and C). To test also the effect of age, we performed the analysis at 8 and 22 weeks. We observed a significant increase in the abundance of type I slow fibres in *Zak^−/−^* female mice when compared with the male counterparts (Fig. 1C). H&E-stained sections confirmed centralized nuclei in both males and females in the *soleus* (Fig. 1D and E), with *Zak^−/−^* males showing higher numbers of regenerating fibres with central nuclei than females in this tissue. Moreover, there was a clear effect of age, with mice showing approximately three times more centrally nucleated fibres at 22 weeks than at 8 weeks (Fig. 1E). Neither fibre atrophy nor central nucleation was identified in the TA muscle from 8-week-old *Zak^−/−^* or control mice (Supplementary Material, Fig. S1). Therefore, to test if ageing exposes any changes also in the pathology free TA muscle, we looked at a later time point. The results showed a significant increase in centrally nucleated fibres in the TA muscle in 14-month-old males and females. Altogether, these results indicate that age is a pathology driver in *Zak^−/−^* mice.

### Regeneration capacity is slightly decreased in Zak^−/−^ mice

**Figure 2:**
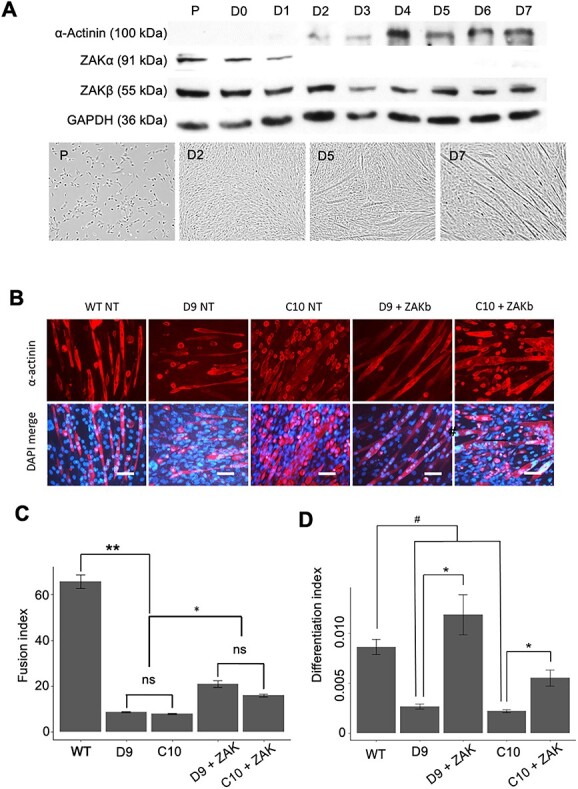
ZAK deficiency affects fusion index of C2C12 myoblasts. (**A**) (Top) Western blots of C2C12 cell lysates from proliferating cells (P) and cells maintained in differentiation medium (DF) up to 7 days (D0 (Day 0)–D7 (Day 7) in DF medium). Immunoblotting was carried out with antibodies against ZAK, which detect ZAKα (91 kDa) and ZAKβ (55 kDa), α-actinin as a marker of differentiation (100 kDa) and GAPDH as loading control. (Bottom) Representative brightfield images of P and differentiating C2C12 cells (note saturation at D2, small myotubes at D5 and long myotubes at D7) depicting the stages of myotube growth over a 7-day period (*n* = 3). Scale bars = 200 μm. (**B**) Representative fluorescence images of C2C12 myoblasts differentiated for 14 days for WT, D9, C10, and D9 and C10 transiently transfected with ZAKβ. Cells were stained for α-actinin (red) and DAPI (blue) (*n* = 3). Scale bars = 200 μm. (**C**) Histogram showing quantification of cell fusion (fusion index) calculated as the percentage of alpha-actinin positive cells with three or more nuclei. KOD9 and KOC10 cells have significantly lower fusion index than WT and rescued cells (Student’s *t*-test; (^*^) *P* < 0.01), (^*^) *P* < 0.05, (^*^^*^) *P* < 0.01). (**D**) Histogram showing quantification of cell differentiation (differentiation index) calculated as the proportion of nuclei within an α-actinin-positive structure to the total number of nuclei in a given field. KOD9 and KOC10 cells have significantly lower fusion index than WT (Student’s *t*-test; (#) *P* < 0.01) and rescued cells (Student’s *t*-test; (^*^) *P* < 0.01).

**Figure 3:**
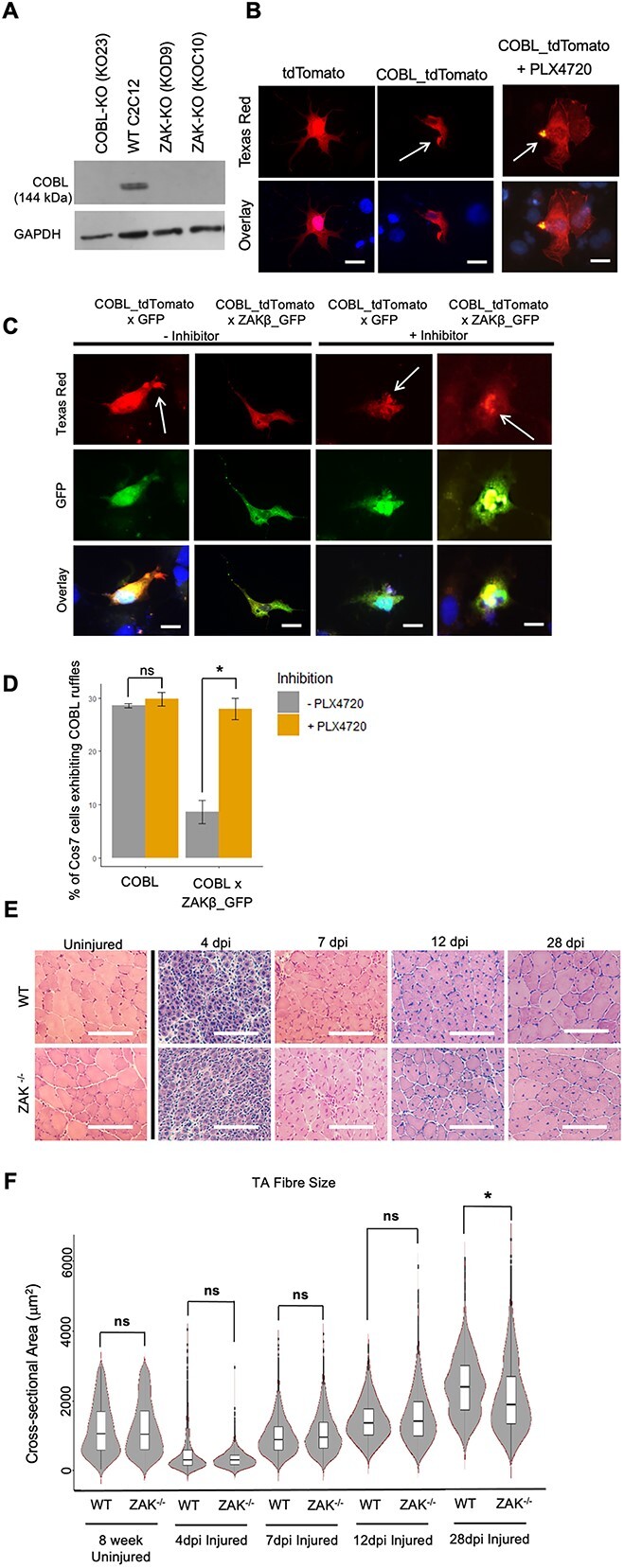
ZAKβ inhibits COBL-mediated ruffle formation in COS7 cells. (**A**) Western blot of COBL KO (KO23), WT C2C12 and ZAK KO (KOD9 and KOC10) cell lines immunostained using anti-COBL antibody. GAPDH used as loading control. (**B**) Representative images of COS7 cells co-transfected with pDEST47-COBL-tdTomato or tdTomato. Note that treatment with the ZAK inhibitor PLX4720 does not affect ruffle formation (white arrows). Scale bars = 25 μm. (**C**) Representative images of COS7 cells co-transfected with pDEST47-COBL-tdTomato and GFP or pDEST47-ZAKβ-GFP in the presence/absence of PLX4720. Arrow indicates the presence of actin ruffles. Scale bar = 25 μm. (**D**) Analysis and quantification of the presence of membrane ruffles in COS7 cells transfected with pDEST47-COBL-tdTomato and either GFP or pDEST47-ZAKβ-GFP. Cells co-transfected with ZAKβ-GFP and COBL-tdTomato displayed significantly fewer ruffles compared with cells co-transfected with GFP and COBL-tdTomato, but the effect of ZAKβ-GFP is prevented by PLX4720 (*n* = 3). Independent *t*-test, (^*^) *P* < 0.01. (**E**) H&E staining of cross sections from WT and *Zak^−/−^* mouse *Tibialis anterior* muscle from 0 to 28 dpi. Note a similar pattern of muscle regeneration subsequent to acute barium chloride injury. Scale bar represents 100 μm. (**F**) Cross-sectional area of 8-week uninjured, 4, 7, 12 and 28 dpi WT and *Zak^−/−^* muscles. Uninjured 8-week muscle, 4, 7 and 12 dpi CSA do not show significant difference between genotypes. Twenty-eight days post-injury muscles show a significantly smaller CSA mean in the regenerating TA from *Zak^−/−^* mice (WT, 2182.42 μm^2^; *Zak^−/−^*, 1875.47 μm^2^), indicating a slight delay in muscle regeneration in *Zak^−/−^* mice (*n* = 5). Independent *t*-test, (^*^) *P* < 0.001.

A role for ZAKβ in muscle regeneration would be consistent with the variable muscle fibre size described in sections from biopsies from human patients (11). To test this, we first analysed the expression of ZAKα and ZAKβ through *in vitro* differentiation of C2C12 myoblasts (Fig. 2). The results showed that while ZAKβ expression is maintained throughout, ZAKα quickly declines as anticipated in differentiation medium (Fig. 2A). We then generated two ZAK-deficient C2C12 lines by CRISPR/Cas9 mutagenesis, hereafter referred to as D9 and C10. Total absence of ZAK in the two independently derived C2C12 clones was confirmed by western blot (Supplementary Material, Fig. S2A and B). The presence of different alleles with disruptive frameshifts was confirmed in both cell lines (Supplementary Material, Fig. S3). As the expression of ZAK overlaps the transition from proliferation to differentiation of C2C12 myoblasts, we tested the fusion and differentiation indices in the ZAK KO cell lines and the parental C2C12 myoblasts (Fig. 2B–D). Quantifications from the D9, C10 and parental lines showed a significant reduction in fusion and differentiation indices in the D9, C10 cell lines. Moreover, these defects were partially rescued by the reintroduction of ZAKβ in both cell lines through transient transfection (Fig. 2B–D). We have previously observed *in vitro* fusion defect phenotypes in cell lines rendered deficient for the ZAK-associated proteins IGFN1 (5) and COBL (18). The implication of the actin nucleating protein COBL in these interactions prompted the hypothesis that the fusion defects in the ZAK-deficient cell lines could be caused by a lack of COBL actin nucleation activity since the requirement of actin remodelling for fusion has been extensively established (e.g. (19–21)). We therefore tested if ZAK*β* modulates the activity of COBL. Surprisingly, both D9 and C10 cell lines show no detectable COBL on western blots (Fig. 3A), suggesting that COBL is either stabilized by ZAKβ or the *COBL* gene is a downstream transcriptional target of ZAKβ. To test COBL activity, we used the ability of COBL to form ruffles in COS7 cells as a read out (Fig. 3B). Ruffles can be identified by COBL concentrating in phalloidin-rich aggregates (18). The results indicate that in cells cotransfected with COBL and ZAKβ constructs, ruffle formation is inhibited (Fig. 3C). Moreover, this inhibition is dependent on the kinase activity of ZAK. Thus, the ZAK inhibitor PLX4720 (22) abolishes the ZAK inhibitory effect on COBL and formation of ruffles is again observed in these cotransfected cells (Fig. 3C and D).

Although the effect of ZAKβ on COBL appears clear in COS7 cells, it is unlikely that a cell fusion defect plays a major role in muscle development *in vivo*, given that *Zak^−/−^* mice develop normally and are overtly indistinguishable from wild-type (WT) littermates (13). Therefore, to test if the *in vitro* observations are relevant to muscle regeneration, we induced acute focal regeneration *in vivo* by intramuscular (IM) injection of BaCl_2_ in the *tibialis* of *Zak^−/−^* and control mice. We choose 8-week-old mice and a pathology free muscle to ensure a robust response to the BaCl2 treatment. Representative H&E images of mid-level sections are shown in Fig. 3E. The results showed an initial phase of acute degeneration and incipient regeneration indicated by the large areas of small centrally nucleated fibres at 4 days post-injection (dpi), followed by a regenerative process from 7 to 28 dpi. Larger fibres lacking central nuclei were evident from 12 dpi (Fig. 3E), but there were no significant differences between samples from *Zak^−/−^* compared with control mice at 12 or 28 dpi (Supplementary Material, Fig. S4). Centronucleated fibres progressively increased in size during the regeneration period (Fig. 3E). As an indirect measure of regeneration capacity, we measured the cross-sectional area of centrally nucleated fibres from the whole *tibialis* (Fig. 3F). The results showed a small but significant reduction in the cross-sectional area of samples from *Zak^−/−^* mice at 28 dpi. We interpret this as the loss of ZAKβ causing a slight delay in muscle fibre regeneration (control mean: 2182.42 μm^2^; *Zak^−/−^*mean: 1875.47 μm^2^; *n =* 5), as there is no difference in fibre cross-sectional area or internal nuclei between genotypes in samples from uninjured young mice (Figs 1G and 3F).

It therefore appears that loss of ZAKβ has a small but measurable effect on the muscle regeneration capacity, independently of confounding factors such as age or pathology that could have influenced this test. However, since COBL appears dispensable for muscle function (23), it seems unlikely that the ZAK/COBL interplay would play a significant role in the pathogenic mechanisms of ZAK deficiency. Therefore, to identify relevant targets of *ZAKβ* kinase activity in skeletal muscle, we next performed a phosphoproteomic screen.

### Phosphoproteomics and bioinformatics identify likely targets and associated disease

**Figure 4:**
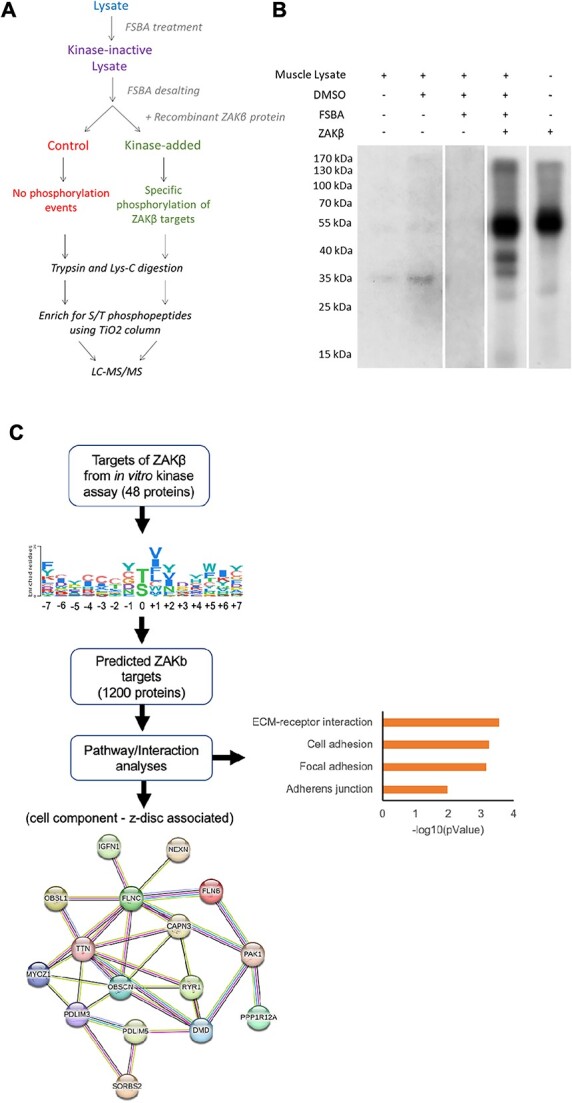
A quantitative phosphoproteomics assay identifies direct ZAKβ substrates. (**A**) Phosphoproteomics workflow utilizing the kinase inhibitor, FSBA. Control samples (red) used to assess background phosphorylation levels of phosphorylated proteins when compared with the samples treated with recombinant ZAKβ (green) after LC–MS/MS. Workflow adapted from Knight *et al.* (49). (**B**) Radioactive *in vitro* kinase assay used as a quality control step to assess kinase activity of recombinant ZAKβ in skeletal muscle lysate. Samples treated with 32P-γ-ATP were separated using SDS-PAGE. Phosphorylated proteins detected using radiography film. Skeletal muscle lysate (lane 1) was treated with either DMSO (lane 2) or FSBA solubilized in DMSO (lane 3). Recombinant ZAKβ was added to skeletal muscle lysate treated with FSBA and desalted using Thermo Fisher Zeba spin desalting 7K column (lane 4). Autophosphorylation activity of the recombinant ZAKβ protein was also assayed (lane 5). (**C**) The phosphoproteomics assay identified 48 significant proteins directly phosphorylated by recombinant ZAKβ. From the PSSM generated, an expanded list of 1200 putative ZAKβ targets was identified. The most significant functions and the Z-disc associated proteins identified by KEGG and STRING analysis, respectively, are shown.

A phosphoproteomics study was undertaken using mouse skeletal muscle extracts and recombinantly produced ZAKβ. Muscle extracts were first treated with 5′-4-fluorosulphonylbenzoyladenosine (FSBA) to irreversibly inhibit all endogenous kinases. Then, active ZAKβ was added to half the samples (treated group), whereas the other half remained untreated (control group) (Fig. 4A, see Materials and Methods for details). In a quality control assay, the FSBA treatment proved effective at inhibiting all endogenous kinases in the muscle lysate, whereas recombinant ZAKβ was able to actively undertake phosphorylation of substrates in the FSBA-treated lysate (Fig. 4B). Enriched S/T phosphopeptides were then analysed by LC–MS/MS and a list of 114 S/T phosphopeptides from 48 individual proteins obtained (Supplementary Material, Table S1). Pathway analyses of this short list (Supplementary Material, Table S1) failed to identify any specific biological process, complex or subcellular compartments (data not shown), but we noted the presence of SYNPO2 (Synaptopodin 2), a protein that interacts with FLNC at the Z-disc (24) and PDLIM5 (PDZ and LIM domain 5), a scaffolding protein that tethers protein kinase D1 at the Z-disc (25,26). To identify further putative ZAKβ targets that may not have been present just in the soluble fraction of the muscle lysate, a position-specific scoring matrix (PSSM) was generated from the 15-mer phosphopeptide sequences identified by LC–MS/MS. The list of direct phosphopeptides was loaded into the PSSMSearch website (http://slim.icr.ac.uk/pssmsearch/) to generate a PSSM as well as a 15-mer consensus sequence (Fig. 4C). The PSSM was then cross-referenced with the online mouse proteome, and 1200 potential ZAKβ targets were identified (Supplementary Material, Table S2). The predicted targets were then subjected to pathway analysis (Fig. 4C, full description in Supplementary Material, Table S3), which identified proteins involved in extracellular matrix–receptor interaction, focal adhesion and cell adhesion as the top enriched KEGG categories (Fig. 4C). The predicted targets included the Z-disc associated proteins FLNC, IGFN1, PDLIM5, MYOZ1 (Myozenin 1) and dystrophin. In a recent study, the optimal substrate specificity for the majority of the human Ser/Thr kinome has been experimentally determined by performing a positional scanning peptide array analysis and a computational approach (27). For ZAK, the top-ranked phosphorylation targets identify myofibrillar myopathy as an associated disease (Supplementary Material, Fig. S5A and Supplementary Material, Table S4). Of the 1555 proteins ranked in the top 10 for ZAK phosphorylation (27), 222 were also identified in our study, including proteins associated with myofibrillar myopathies such as FLNC, BAG3 (BCL2-binding athanogene 3) and TTN (Titin) (Supplementary Material, Fig. S5B and Supplementary Material, Table S5).

Recently generated RNAseq data from *Zak^−/−^* and control mice (accession number PRJNA816072, (13)) also identified extracellular matrix–receptor interaction and focal adhesion as the top pathways in the differentially expressed genes (DEGs) obtained from the pathology free TA muscle (Supplementary Material, Fig. S6 and Supplementary Material, Table S6). These same pathways also appear to be highly enriched in pathological *soleus* muscle (Supplementary Material, Fig. S6 and Supplementary Material, Table S7). In addition, enrichment of the same top KEGG pathways was obtained from the list of DEGs from microarray analysis of ZAK-deficient patient muscle biopsies (11). Thus, the results from the phosphoproteomics as well as the transcriptional profiles from *Zak^−/−^* mice and patients suggest that focal adhesion components are both substrates and downstream transcriptional targets of ZAKβ. Table 1 summarizes the list of genes contributing to focal adhesion for each of the above studies, in which filamins, integrins, collagens and thrombospondins appear as common hits. A role for ZAKβ in cell adhesion in muscle would be consistent with the reported relocalization of ZAKβ to stress fibres emanating from focal adhesions after an acute compression stimulus (13). As a generic test of cell adhesion and migration, we performed a wound healing assay on the ZAK-deficient and control cell lines (Supplementary Material, Fig. S5A). For this, the distance travelled by the sheet migration after creating a gap in the cell monolayer was measured at regular intervals for a 24-h period. The results consistently showed smaller distances of sheet migration in the D9 and C10 cell lines compared with C2C12 control (Supplementary Material, Fig. S5B). To account for the effect of cell proliferation rates, the assay was repeated using a live-cell imaging microscope that allowed individual cell tracking and cell division time measurements. Cell migratory speed was calculated by the total distance travelled divided by time over a 30-h period. The results showed that D9 and C10 migratory speeds are significantly lower than the C2C12 control (Supplementary Material, Fig. S5C), whereas cell division times for C212, D9 and C10 were 20.6, 27.4 and 22.3 h, respectively. We therefore conclude that the ZAK-deficient clones D9 and C10 have lower migration speeds than the C2C12 parental cell line.

**Table 1:** Summary list of focal adhesion genes identified in phosphoproteomics and RNAseq data from mouse and human

Putative ZAKβ substrates (from phospho-proteomics logo)	Mouse *soleus* DEGs (RNAseq data)^a^	Mouse TA DEGS (RNAseq data)^a^	Skeletal muscle DEGs (patient RNAseq data)^b^	Common hits
ARHGAP5	AKT3	FLNC	COL1A1	Filamins
BCAR1	CAV1	FN1	COL1A2	Integrins
BRAF	CAV2	ITGA9	COMP	Collagens
COL4A2	CAV3	LAMC2	HGF	Thrombospondins
COL6A1	COL1A1	MYL12A	IGF1	
COL6A5	COL4A1	THBS2	TTGA10	
COMP	COL4A2	THBS3	ITGA3	
DIAPH1	COL6A6	THBS4	ITGA9	
FLNA	COMP	TNC	ITGB6	
FLNB	EGF	TNXB	ITGB8	
FLNC	FLNB		FLNC	
FN1	FLT1		MYLK4	
ITGAV	FN1		THBS2	
ITGB7	IGF1		THBS4	
LAMA3	ITGA3		TNC	
LAMA5	ITGA6			
LAMB1	ITGA7			
MYLK	ITGA9			
PAK1	ITGB1			
PARVA	KDR			
PIP5K1B	LAMA5			
PP1R12A	LAMB1			
SHC3	LAMC2			
THBS3	MYL10			
THBS4	MYL2			
	MYL9			
	MYLK			
	MYLK3			
	MYLPF			
	PDGFRB			
	SHC2			
	THBS4			
	TNC			
	VEGFA			
	VTN			

^a^Patient RNAseq data from Nordgaard *et al.* (13). ^b^RNAseq data from Vasli *et al.* (11).

From the table of putative ZAKβ targets (Supplementary Material, Table S3), Filamin C (*FLNC)* attracted our attention because of its role in cell adhesion through its interactions with the transmembrane receptor β1-integrin (28,29) and components of the dystrophin–glycoprotein complex (28). Moreover, SYNPO2 was identified as a direct phosphorylation target of ZAKβ at S540 (Supplementary Material, Table S1). Since SYNPO2 is involved in the turnover of FLNC through the chaperon-assisted selective autophagy mechanism (CASA) (30), we decided to investigate FLNC expression both in the mouse KO and in the biopsy of a ZAK-deficient patient.

### FLNC turnover is disrupted in some fibres from ZAK-deficient muscle

We studied FLNC expression on *soleus* sections of 8-week-old mice. Both males and females show a high percentage of fibres with strong anti-FLNC antibody reactivity (Fig. 6A). FLNC appears highly concentrated in some fibres from the *Zak^−/−^* mouse, which are not detected in control sections (Fig. 6B). The percentage of fibres in the *soleus* showing these highly immunoreactive fibres is significantly reduced at 22 weeks in *Zak^−/−^* males and females (Fig. 6B). We identified BAG3, the core cochaperone in the CASA mechanism (30,31), showing similar distribution in the same FLNC-positive fibres in the *Zak^−/−^ soleus* sections (Fig. 6C). Interestingly, neither *Flnc* nor *Bag3* transcript levels are differentially expressed in the mouse *soleus* RNAseq data (Supplementary Material, Fig. S7), suggesting that the increased signal on immunofluorescence is because of the CASA pathway being unable to cope with the FLNC turnover in some fibres. Since CASA is a tension-elicited pathway (31), we decided to test if mechanical stress would exacerbate these observations as predicted. For this, we tested the ability of ZAK-deficient *soleus* muscle to respond to chronic overload. We applied synergistic ablation to 30-week-old female mice and surgically removed the *gastrocnemius* muscle from one of the hind limbs. We then quantified the fibres showing abnormal reactivity to FLNC antibodies. The results showed an acute and significant difference in the overloaded muscle in *Zak^−/−^* compared with control (Fig. 6D and E). Thus, while the control showed up to 3% reactive fibres in the overloaded muscle from <1% in the sham leg, the *soleus* from *Zak^−/−^* mice showed an average of 15% reactive fibres in the overloaded muscle compared with 3% in the sham. We also confirmed the presence of myotilin, another myofibrillar myopathy marker (32), in the FLNC-positive fibres (Supplementary Material, Fig. S8). Thus, despite the progressive adaptive changes that soleus goes through described in Figure 1, it remains more susceptible to mechanical stress in Zak−/− mice compared with age-matched controls. Intriguingly, the percentage of fibres in the soleus showing aberrant distribution of FLNC is significantly reduced at 22 weeks in males and females (Fig. 6B), indicating that the age-dependent adaptive changes in the soleus allow this tonic muscle to cope better with mechanical loading. As expected, spared TA muscles did not show FLNC immunoreactive fibres at 8 weeks (Supplementary Material, Fig. S9).

**Figure 5:**
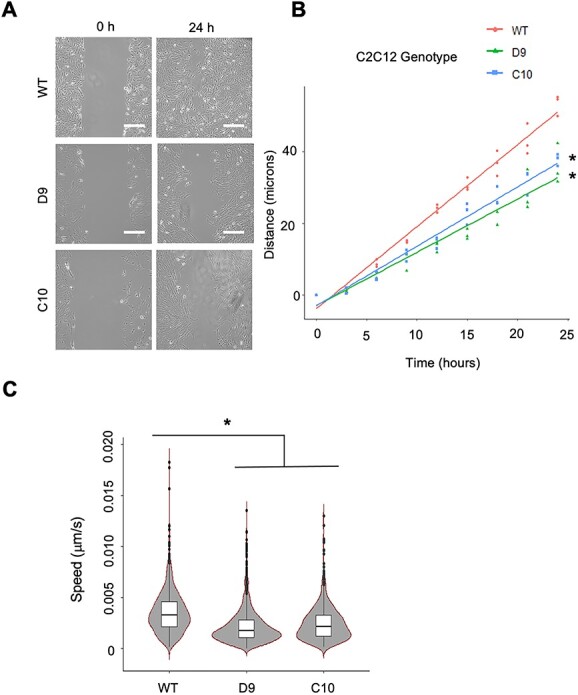
ZAK-deficient C2C12-derived cell lines display a reduced migratory ability in culture. (**A**) *In vitro* scratch wound assay of WT and ZAK KO C2C12 cell lines (D9 and C10). Representative brightfield images taken at 0 h and upon completion at 24 h. Scale bars = 25 μm. (**B**) Linear plot of the distance travelled to close the wound over a 24-h period. Measurements taken at 3-h intervals with three replicates per cell line per time point. Within the time frame, WT cells showed full closure, whereas D9 and C10 cell lines failed to close the gap (*n* = 3). Independent *t*-test, (^*^) *P* < 0.01. (**C**) Distribution plot of every cell track as measured by the total distance travelled by individual cells divided by time for C2C12 (WT), D9 and C10 cell populations, as indicated. Independent *t*-test, (^*^) *P* < 0.01.

**Figure 6:**
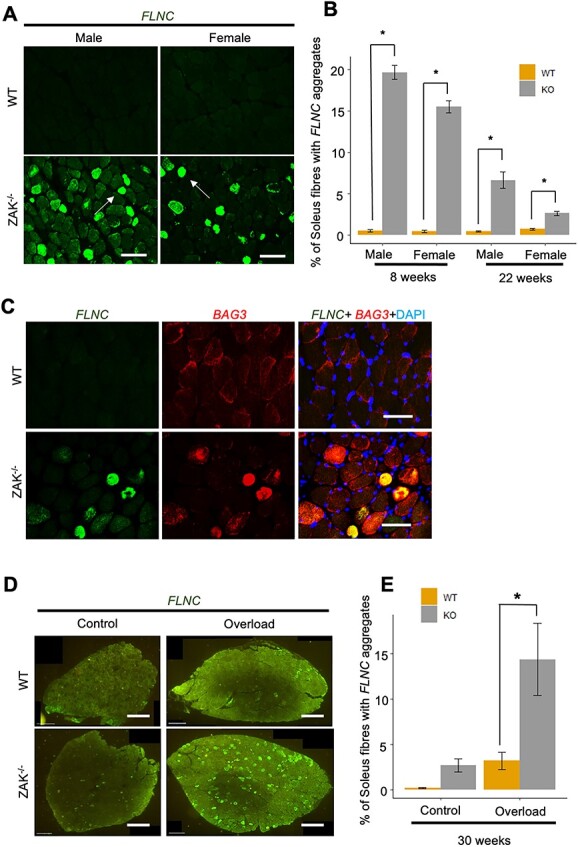
Accumulation of the actin cross-linking protein FLNC in ZAK deficient muscle fibres. (**A**) Immunofluorescence images of *soleus* muscle cross sections of 8-week-old male and female, control and *Zak^−/−^* mice stained with anti-FLNC (green). FLNC accumulation is observed only in the *Zak^−/−^* mouse (white arrows). Scale bar represents 250 μm. (**B**) Quantification of the percentage of 8- and 22-week-old male and female entire *soleus* muscle fibres exhibiting FLNC aggregations (*n =* 3). Independent *t*-test; (^*^P < 0.05). FLNC immunoreactive fibres were observed to significantly decrease with age and sex. Independent *t*-test (^*^P < 0.05). (**C**) Representative immunofluorescence images of FLNC (green) and BAG3 (red) in 8-week WT and *Zak^−/−^ soleus* muscle. Scale bars = 100 μm. (**D**) Representative fluorescence images of FLNC (green) in 4-month-old female WT and *Zak^−/−^ soleus* muscle cross sections of sham (control) and experimental leg muscles from the same mouse (*n =* 6). Note the increment of highly reactive fibres to anti-FLNC antibodies and their different patterns in the overloaded *Zak^−/−^ soleus* muscle Scale bars = 500 μm. (**E**) Quantification of the positive FLNC fibres observed across the whole muscle cross section as a percentage of total fibres. *Zak^−/−^* overloaded leg showed a significant increase in FLNC-positive fibres when compared with the overloaded leg of the WT mouse (*n* = 6). Independent *t*-test (^*^) *P* < 0.05. The percentage of these fibres in the controls are consistent for this age group.

**Figure 7:**
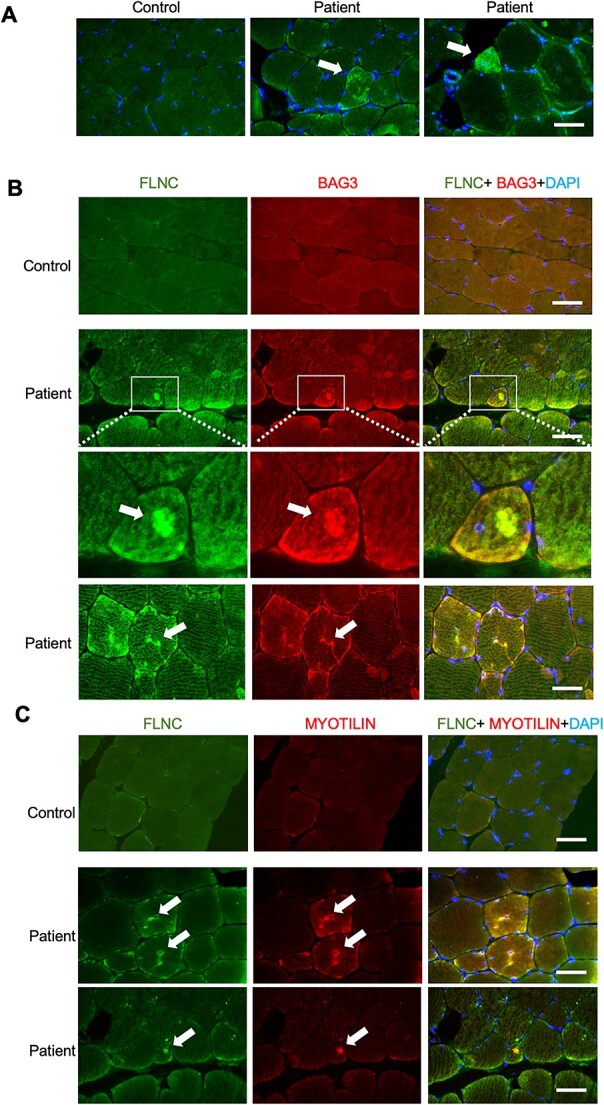
Aggregation of FLNC, BAG3 and myotilin in sections from a ZAK-deficiency biopsy. (**A**) Immunofluorescence with anti-FLNC (green) of skeletal muscle sections from biopsies from a control and a ZAK-deficient patient. Irregular FLNC expression is observed only in the patient (white arrow). Scale bar represents 100 μm. (**B**) Co-immunofluorescence images from skeletal muscle sections from a control and a *ZAK^−/−^* patient stained with anti-FLNC (green) and anti-BAG3 (red) antibodies. FLNC and BAG3 identify the same fibre and subcellular accumulation only in the patient (white arrow). Scale bar represents 100 μm. The inset showing a positive fibre (white box) has been magnified below the original images. (**C**) Co-immunofluorescence images from skeletal muscle sections from a control and a ZAK-deficient patient stained with anti-FLNC (green) and anti-myotilin (red) antibodies. Note that these are consecutive serial sections from those presented in (B). FLNC, BAG3 and Myotilin show irregular expression in the same fibres of the patient (white arrows). Scale bar represents 100 μm.

To test the relevance of the findings above in the ultra-rare recessive human condition associated with ZAK deficiency, we analysed a biopsy sample from a previously described congenital myopathy patient (11). This patient carries a truncating variant in the *ZAK* gene in homozygosity (c.490?491delAT, p.Met164fs^*^24, using reference sequence NM_133646) (11). We identified a small number of fibres that showed very high reactivity with FLNC antibodies in strong accumulations (Fig. 7A). Serial sections and double immunofluorescence showed that the same fibres stained by BAG3 and FLNC were also positive for myotilin (Fig. 7B and C).

**Figure 8:**
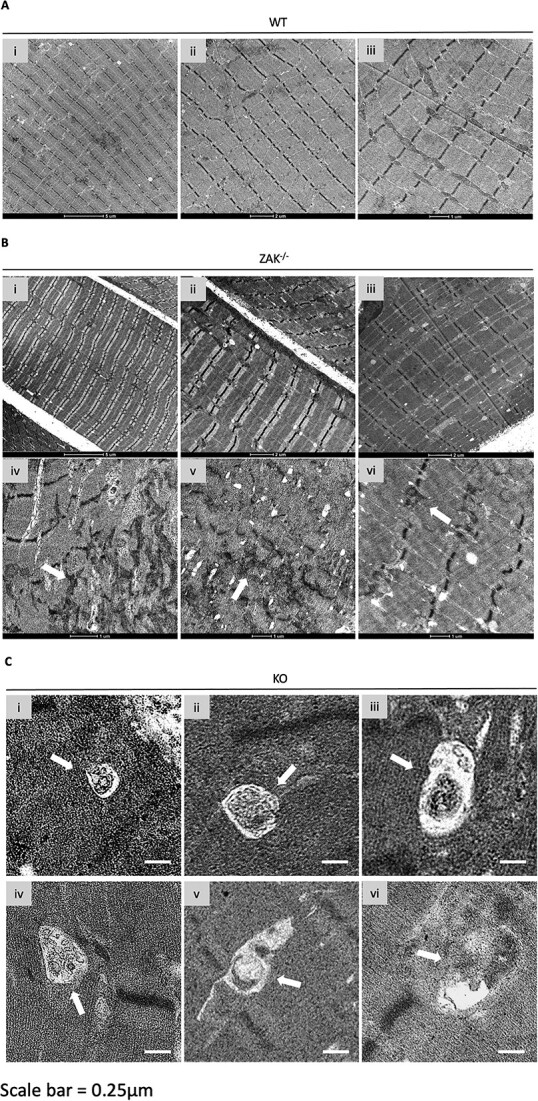
Electron microscopy analysis of *Zak^−/−^* mouse and control *soleus* muscle. (**A**) (i–iii) Panoramic images of 8-week-old WT *soleus* muscle. Scale bars values detailed on image. (**B**) (i–iii) Representative images of fibres from the *Zak^−/−^ soleus* showing normal preservation of sarcomeric structure. (iv–vi) Examples of Z-disc and overall sarcomeric disorganization identified only in *Zak^−/−^* mice (*n =* 3). Scale bars values detailed on image. Arrows point at apparent dissolution of the Z-disc. (**C**) (i–vi) Vacuoles (white arrows) containing membranous and electron dense material were identified only in samples from *Zak^−/−^* mice (*n =* 3). Scale bar represents 250 nm.

Thus, although the majority of the muscle remains apparently normal, expression of FLNC and other markers suggest that ZAK deficiency shows features of a mild myofibrillar myopathy. To look for evidence of this at an ultrastructural level, we performed electron microscopy of *soleus* muscle on 8-week-old animals (Fig. 8). Overall, *soleus* samples from *Zak^−/−^* mice (*n =* 3) presented a normal ultrastructure indistinguishable from control muscles. Granulofilamentous material or nemaline rods described in myofibrillar myopathy (e.g. (33)) were not found. There was limited evidence of myofibrillar and Z-disc disorganization (Fig. 8B), which was not detected in the age- and sex-matched controls (Fig. 8A). Moreover, large vacuoles containing membranous material were observed, likely reflecting disrupted autophagy in isolated fibres (Fig. 8C). We did not attempt to quantify these observations, as sampling of the muscle using this technique was deemed insufficient.

### Aged ZAKβ zebrafish KO show reduced locomotor performance

**Figure 9:**
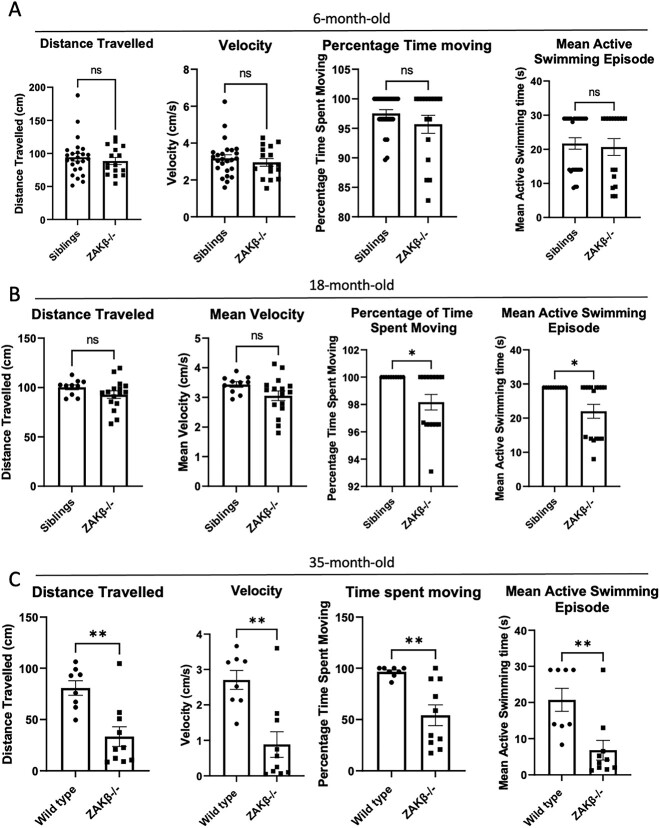
Comparison of key swimming attributes between 6/18/35-month-old *Zakβ^−/−^* zebrafish and *Zakβ^+/−^* siblings. Analysis of swimming attributes of 6-month-old (**A**), 18-month-old (**B**) and 35-month-old (**C**) *Zakβ^+/−^* X *Zakβ^−/−^* zebrafish. The parameters measured are ‘distance travelled’ (cm), ‘velocity’ (cm/s), ‘percentage of time moving’ and ‘mean length of time for each swimming episode(s)’. Each data point is an individual recording. The bar chart and error bars are mean ± standard error of the mean. An unpaired, two-tailed *t*-test was the statistical test performed. Ns = not significant. ^*^P ≤ 0.05. Twelve WT and eight *Zak^−/−^* zebrafish had recordings taken, providing 24 data points for WT and 16 data points for *Zak^−/−^*.

We next tested the effect of loss of *ZAKβ* in an independent animal model. In zebrafish, *Zakα* and *Zakβ* are encoded by different genes. We generated a homozygous loss of function mutant line by CRISPR/Cas9 mutagenesis of exon 2 of the *Zakβ* gene (ENSDARG00000044615.8), following the strategy described in Materials and Methods. The mutation is a 33 bp insertion with an in-frame stop codon upstream of the kinase domain (Supplementary Material, Fig. S10). We then performed a number of locomotor tests using the experimental platform previously described (34) in conjunction with an in-house developed software to track the movement of individual zebrafish. Four parameters were measured: the distance travelled, percentage of time spent moving, swimming episode duration and mean velocity. These parameters were measured on independent cohorts of 6- and 18-month-old fish. The results summarized in Figure 9 show no significant differences are observed in 6-month-old fish, whereas the 18-month-old *Zakβ*^−/−^ fish show a trend in lower distance travelled and mean velocity and a significant difference with controls for lower percentage of time spent moving and mean active swimming episode. To confirm this trend, we tested 35-month-old *Zakβ*^−/−^ fish against same-age WT controls that had been kept in different tanks. The results show that differences with controls are exacerbated by age, with all parameters measured showing lower performance for the 35-month-old *Zakβ*^−/−^ fish, noting that the results for these particular groups do not account for any potential variability provided by the tank environment.

**Figure 10:**
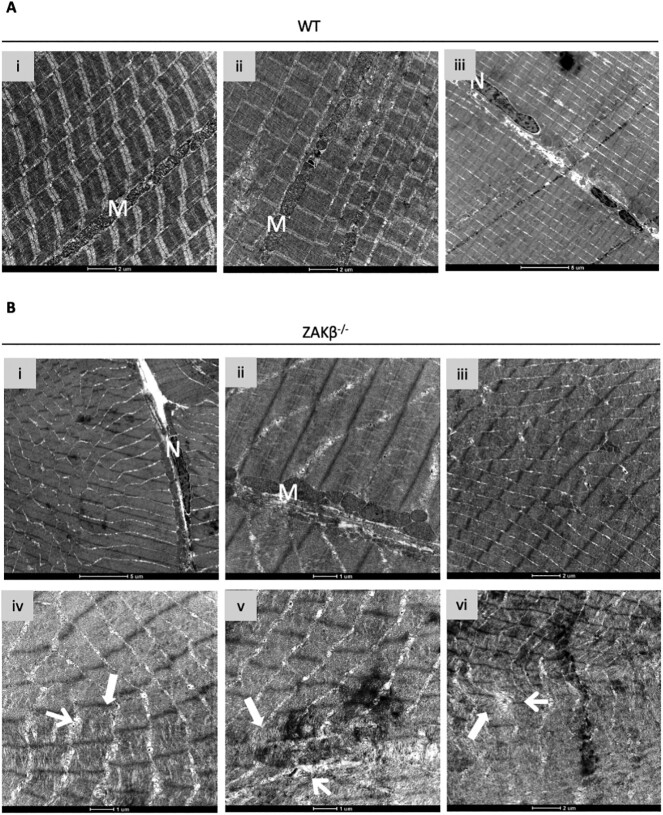
Electron microscopy analysis of *Zak^−/−^* zebrafish skeletal muscle. (**A**) (i–iii) Panoramic images of WT zebrafish skeletal muscle. N indicates nucleus; M indicates mitochondria. (**B**) (i–iii) Representative images of fibres from the *Zakβ^−/−^* zebrafish skeletal muscle showing normal preservation of sarcomeric structure. (iv–vi) Areas of skeletal muscle showing partial loss of myofibrillar and sarcomeric organization were identified only in samples from *Zakβ^−/−^* zebrafish (*n =* 3).

The 35-month-old zebrafish were also used for ultrastructural analysis of skeletal muscle (Fig. 10). Broad views of electron microscopy images showed no differences with controls (Fig. 10A), with both genotypes showing well defined sarcomeres, mitochondria and nuclei. However, similar to the observations in mice, sporadic fibres showing apparent dissolution of the filamentous material were detected only in the samples from *Zakβ*^−/−^ fish (Fig. 10B).

## Discussion

Loss of function of *Zak* causes a mild disease in mice compared with the congenital myopathy condition described in humans (11,35). *Zak*^−/−^ mice do not show any overt difference to controls, including in behavioural tests (13). Common findings such as fibre atrophy and predominance of slow type fibres nonetheless suggest that analysis of the mouse pathogenesis can shed light on the human disease. Within the hindlimb, the tonically active *soleus* shows pathology in young mice, whereas *tibialis* and other muscles are spared. Age is a driver of pathology. Thus, in old mice, the proportion of centrally nucleated fibres increases in the *soleus*, whereas the spared *tibialis* begins to show similar changes. There is also a progressive shift with age in *soleus* to type I fibres expressing the slow myosin heavy-chain subunit. This fibre type conversion is likely adaptive, as ageing females show more type I fibres and less pathology than the age-matched males, suggesting that the higher fatigue-resistant profile of the female *soleus* contributes to ameliorate the pathology. In conclusion, in the mouse, tonically active muscles appear more susceptible to the loss of ZAKβ, consistent with contraction stimuli being a trigger of ZAKβ signalling (13).

ZAKα and ZAKβ are expressed in proliferating C2C12 myoblasts and at the start of myoblast fusion. This prompted an *in vitro* test of myoblast fusion and differentiation, which showed a fusion defect in C2C12-derived ZAK KO cell lines. We hypothesized that disruption of COBL actin nucleation activity in the ZAK KO cell lines underlied the fusion defect. Unexpectedly, expression of COBL was blunted in two independently generated ZAK-deficient clones. We could not confirm COBL expression *in vivo* because antibodies performed poorly on muscle extracts, but a critical role for ZAK or COBL in myoblast fusion *in vivo* can be ruled out since both ZAK and COBL KO mice have a normal muscle development (13,23). Moreover, ZAKβ is not a key player of muscle fibre regeneration of adult muscle either, as in *Zak*^*−/*−^ mice, there is a small size effect on the speed of regeneration following injury and full regeneration is eventually achieved. This small delayed regeneration phenotype could be exacerbated at older time points since regeneration capacity declines with age (36). Therefore, ZAKβ function appears critical for muscle function but not for muscle development or regeneration.

To identify other ZAKβ targets that may contribute to the pathogenesis, we performed a phosphoproteomic screen. The phosphoproteomics assay as well as transcriptional profiles in patients (11) and mice (13) identified components of cell/focal adhesion as the most represented, which prompted the hypothesis that ZAKβ may regulate the turnover of cell adhesion factors. To test this hypothesis, we decided to focus on FLNC for several reasons. FLNC plays an important role linking the sarcolemma to the extracellular matrix (28,29) and interacts with SYNPO2, a direct substrate of recombinant ZAKβ and component of the CASA protein turnover mechanism (30). Moreover, we note that *Zak^−/−^* mice show similar but much less severe pathology to that of *Ky^−/−^* mice (37,38). For example, in *Ky^−/−^* mice the shift to type I fibres in *soleus* is extreme and within 3 months, they make up 100% of this tissue (6), with FLNC showing strong mislocalization patterns (39). In *Zak^−/−^* mice, we confirmed the presence of fibres showing aberrant accumulation of FLNC in the *soleus* in males and females. Interestingly, as the *soleus* shifted towards type I fibres with age, the proportion of fibres with irregular FLNC expression decreased, with females showing less aberrant expression than males. Since FLNC is a well-characterized client of CASA (31), we interpret our results as deficient turnover of FLNC through the CASA mechanism (30). Under this hypothesis, in the absence of ZAKβ*,* costameric*/*transmembrane complexes are less able to cope with mechanical stress, overwhelming the CASA mechanism and leading to the accumulation of damaged FLNC in some fibres. Accumulation of the core CASA cochaperone BAG3 (31) in the same fibres that show irregular FLNC expression supported that notion. Furthermore, increased endogenous loading by partial ablation of the *gastrocnemius* in the hind limb caused a dramatic increase in the accumulation of fibres showing irregular FLNC expression in the *Zak^−/−^ soleus*, indicating that ZAKβ signalling is necessary for a normal physiological response to sustained overloading. Despite the inherent limitations of the material, similar FLNC/BAG3 immunoreactive fibres were also detected in muscle sections from a ZAK-deficient patient biopsy. Moreover, Myotilin, a FLNC partner at the Z-disc (40) that underlies myofibrillar myopathy (32), accumulates in the same FLNC-positive fibres in mice and humans. It thus appears that ZAKβ signalling is required for the turnover of certain proteins and those identified here, FLNC, BAG3 and Myotilin, are implicated in myofibrillar myopathies (41).

Electron microscopy views confirmed normal sarcomeric ultrastructure in *soleus* sections from the mutant and control mice. Rare examples of Z-disc disorganization or presence of large vacuoles with dislocated membranous content were detected only in samples from *Zak^−/−^* mice. Of note, rimmed vacuoles, autophagic in nature and often reported in myofibrillar myopathies (41), were also previously detected in biopsies from ZAK-deficient patients (11). We generated *Zakβ^−/−^* zebrafish to test the presence of the above features in an independent model. Electron microscopy showed normal preservation of organelles and sarcomeric organization, with sporadic evidence of myofibrillar disorganization in samples from 35-month-old fish, which were not found in age- matched controls. In all tests performed, *Zakβ^−/−^* zebrafish showed no phenotype at 6 months, but locomotor performance decreased with age, indicating that ZAKβ signalling also contributes to preserving normal muscle function in zebrafish. In summary, whereas ZAKβ loss of function in humans causes measurable muscle defects from birth (11), most phenotypic observations in smaller animal models appear with age and the ultrastructural hallmarks of myofibrillar myopathy, namely, Z-disc streaming, granulofilamentous/filamentous sarcoplasmic inclusions or large autophagic vacuoles (42,43), are absent or marginally present in the animal models.

In the majority of myofibrillar myopathies, the mutant protein is a major component of the proteomic content of the aggregates they appear to cause (44). This is not a plausible mechanism for ZAK deficiency, as the loss of function *ZAK* mutations so far reported a result in total absence of protein (11,13). However, recessive loss of function mutations in the *KY* gene also result in mislocalized FLNC expression in patients (8,9) and mice (39), indicating that formation of aggregates driven by the mutant protein is not a universal mechanism in myofibrillar myopathies (45). The accumulation of mislocalized FLNC and BAG3 in highly reactive fibres reported here may potentially trap these proteins away from the Z-disc. Sequestration of FLNC or BAG3 has been proposed as a mechanism for myofibrillar myopathy whereby their accumulation depletes them from the Z-disc, compromising function and structural integrity in these fibres (46,47). Our data suggest that a similar mechanism may contribute to the muscle pathology of ZAK deficiency in mice and humans.

## Materials and Methods

### Cell culture

Murine C2C12 myoblasts were cultured in growth medium; Dulbecco’s Modified Eagle’s Medium (DMEM, Gibco; S41966-029) supplemented with 10% fetal bovine serum (Gibco; S10270), penicillin and streptomycin (P/S, Gibco; S15140 (100 U/ml)). Cells were maintained in a humidified cell incubator at 37°C with exposure to 5% CO_2_. C2C12 myoblasts were differentiated into myotubes through the replacement of growth medium with differentiation medium; DMEM +2% horse serum + P/S (HS, Gibco; 26 050). Media were replaced daily and left to differentiate for 7 days.

For transfection, cells were seeded in either 6-well or 24-well plates in growth medium. Transfections were performed according to manufacturer’s guidelines when cells reached 80% confluency. The TransIT-X2 (Mirus Bio; MIR 6000) transfection reagent was used in the transfection of both C2C12 and COS7 cells. After 48 h, cells were fixed, clonally selected or processed for differentiation analyses. For fixation, transfected cells were washed twice with pre-warmed 1x PBS and fixed with either 4% PFA for 10 min or ice-cold acetone: methanol (1:1) for 20 min. After fixation, cells were washed twice with 1x PBS and then with ddH_2_O ready for immunofluorescence analyses.

### Western blot

For protein extraction from cells, cells placed on ice and washed twice in ice-cold 1x PBS. Cells were lysed following the addition of minimal RIPA buffer (Sigma; R0278) + protease inhibitor cocktail (PI, Sigma; P8340) on ice. Lysed cells were subjected five times to 5-s pulses of sonication with 1-min rest on ice between each pulse. Cell lysate was centrifuged at 15 000*g* for 15 min at 4°C. The supernatant was collected and stored for processing. Samples were mixed with 4x NuPAGE LDS buffer (Invitrogen; NP0008) and 10x NuPAGE Reducing Agent (Invitrogen; NP004) and boiled for 10 min before being resolved by SDS-PAGE and transfer onto nitrocellulose membranes. Membranes were blocked in PBS-T (1x PBS supplemented with 0.2% v/v Tween-20) + 5% milk followed by overnight (ON) incubation with primary antibodies. The following antibodies were used for western blot in this study: 1:2500 rabbit anti-ZAK (Proteintech. 14945-1-AP), 1:500 mouse anti-sarcomeric α-actinin (Abcam, EA-53), 1:20 000 anti-GAPDH-HRP (Sigma, G9295) incubated in blocking buffer ON at 4°C. Membranes were then washed in PBS-T three times and incubated for 1 h with HRP-conjugated secondary antibodies: anti-rabbit IgG (Santa Cruz Biotechnology, sc-2030 1:10 000) and anti-mouse (Santa Cruz Biotechnology, sc-20051:10 000), followed by further three PBS-T washes. Detection was performed using Immobilon Western Chemiluminescent HRP Substrate (Millipore, P09718). Bands were detected using autoradiography film (Santa Cruz Biotechnology, sc-210696) and developed using an Xograph film developer.

### Generation of ZAK KO C2C12 cell lines via Crispr/Cas9

Pre-designed vectors were purchased from VectorBuilder. ZAKβ CRISPR/Cas9 KO plasmid (VectorBuilder, VB191031-4552uqf) was obtained for CRISPR/Cas9 targeting. Specific target sites within exons 2 and 3 of ZAKβ were identified and targeted using two gRNAs:

• gRNA1 = GGAGTGTGTATCGAGCCAAA

• gRNA2 = CCAACTATGGCATCGTCAC

SacII-linearized ZAKβ CRISPR/Cas9 KO vector (VB191031-4552uqf) was transfected into WT C2C12 cells using TransIT-X2 system as described above. After 48 h, the GM was replaced with GM + neomycin (1.5 mg/ml). Transfected cells were then maintained at 37°C in a humidified 5% CO_2_ incubator. Media was changed every 2 days until the formation of resistant colonies. Resistant colonies were re-seeded in a 96-well plate via serial dilution, to identify individual clones. Cells were observed and wells in which single colonies formed were selected for expansion, differentiation and analysis. Surviving clones were sequenced, and the nature of the mutation was determined.

### Immunofluorescence

Snap-frozen muscle tissue was first sectioned using a cryostat machine to a thickness of 10 μm, and slides were stored at −80°C. Slides were removed from −80°C and left to thaw at RT until condensation had evaporated. Blocking was performed in 4% bovine serum albumin (BSA) in 1x PBS. Sections were incubated with the following primary antibodies ON at 4°C; 1:100 anti-MyHC1 (A4.840, DSHB), 1:100 anti-MyHC2a (SC-71, DSHB) 1:150 anti-FLNC (RR90, gift from Peter Van der Ven), 1:150 anti-BAG3 (10599-1-AP, Proteintech) and 1:80 anti-MYOT (RS034, Novocastra) in blocking solution. Sections were subject to 3x 5-min washes in 1x PBS and incubated with the following secondary antibodies at RT for 1 h; 1:150 goat anti-mouse IgG-FITC (F9006, Sigma), 1:150 goat anti-mouse IgM-TRITC (SAB3701196, Sigma), 1:150 goat anti-mouse IgA-FITC (F9006, Sigma), goat anti-rabbit IgG-alexafluor594 (R37117, Invitrogen) and goat anti-mouse IgG-alexafluor594 (R37121, Invitrogen). Sections were again subject to three times 5-min washes in 1x PBS. Slides were mounted with Mowiol mounting medium with DAPI. A coverslip was added and compressed avoiding the formation of air bubbles.

Fixed cells were permeabilized by incubation with 4% BSA in 1x PBS supplemented with 0.1% triton-X100 for 30 min. This was followed with blocking using 4% BSA in 1x PBS for 1 h and ON incubation with 1:150 anti-α-actinin (EA-53, Sigma) at 4°C. Cells were incubated with goat anti-mouse IgG-alexafluor594 (R37121, Invitrogen) for 2 h following 3× PBS washes. Cells were further washed 3× with 1× PBS, followed by a final wash with ddH_2_O. Cells cultured on coverslips were mounted onto slides with Mowiol + DAPI. For cells cultured directly onto plastic, the bottom of the plastic dish was removed by cutting it using specialized laser equipment in order to isolate the bottom of the well and mounted with a coverslip using Mowiol + DAPI.

### BaCl_2_ treatment

BaCl_2_ was selected as the method of injury as the levels of inflammatory cells return to basal levels quicker when compared with notexin or freezing injury models (50). WT and Zak^−/−^ mice (8 weeks) were anaesthetized with 2% isoflurane. To injure the leg, IM injections of barium chloride (BaCl_2_; 1.2% in sterile saline, 30 μl) were delivered to the *tibialis* under general anaesthesia. The *tibialis* muscle from the contralateral leg remains uninjured. Five replicates were performed per condition. Injections were performed on control and Zak^−/−^ mice. Three uninjured mice were used as non-injured controls. After the procedure mice were left for 4, 7, 12 and 28 dpi to recover. Mice were euthanized by cervical dislocation, and the injured *tibialis* was isolated and snap-frozen in isopentane for sectioning. Cross sections of *tibialis* were obtained at proximal, central and distal levels along the long axis of the muscles, H&E stained and imaged as described.

### Synergistic ablation

WT and Zak^−/−^ mice (30 weeks) were anaesthetized with 2% isoflurane. To mechanically overload the *soleus* muscles, the distal third of the *gastrocnemius* muscle is removed from one leg, leaving the *soleus* muscle intact. The *gastrocnemius* muscle from the contralateral leg remains intact and acts as a control. After the procedure mice were left for 2 weeks in individually housed cages. Immediately after the surgical procedure and 6, 12, 18 and 24 h after the surgery, each mouse is injected with analgesics (buprenorphine—Temgesic, 0.05–0.1 mg/kg). In addition, the mice are injected with carprofen immediately after surgery (when still anesthetized) and 24 h after surgery. After 14 days, the mice were euthanized by cervical dislocation and the *soleus* (sham and overloaded) muscles were isolated and embedded in OCT compound and frozen in liquid nitrogen–cooled isopentane and then stored at −80°C for further analysis.

### Skeletal preparation

Zak^−/−^ and WT mice were sacrificed at 28 weeks old, respectively, and fixed in 10% neutral buffered formalin (Sigma-Aldrich, HT501128) for 48 h. Samples were rinsed with ddH_2_O and left for 24 h with gentle shaking. Both samples were post-fixed in 70% ethanol for 5 days, with the ethanol solution refreshed daily. Following post-fixing, specimens were dissected. This process involved removal of the skin, eyes, visceral organs and adipose tissue between the scapulas and behind the neck. Specimens were placed in two changes of 95% ethanol ON at RT to dehydrate and further fix the remaining tissue. The ethanol solution was replaced with 100% acetone for a further 2 days to fix the specimens and remove excess adipose tissue. Specimens were stained with Alcian blue stain solution (0.02% (w/v) Alcian blue 8GX (Thermo Fisher; 15432949), 70% EtOH, 30% glacial acetic acid) for 4 days. Samples were washed with ethanol/glacial acetic acid (7:3) for 1 h and fixed in 100% ethanol ON at RT. Specimens were rinsed in ddH_2_O for 3 days before being incubated with 1% trypsin (in ddH_2_O containing 30% sodium borate) for 24 h. Digested specimens were subjected to staining with Alizarin red stain (0.005% (w/v) Alizarin red (Alfa Aesar; 11 319 707) in 0.5% KOH (w/v)) for 48 h. Specimens were transferred to 1% KOH clearing solution for 2 days followed by decreasing gradients of 1% KOH to glycerol to further clear the specimens, i.e. 3:1, 1:1, 1:3 and 100% glycerol at 2 days per step. Specimens were stored in 100% glycerol prior to imaging.

### Immunohistochemistry

Snap-frozen muscle tissue was first sectioned using a cryostat machine, and slides were stored at −80°C. Sections were cut at 12 μm. Slides were removed from −80°C and left to thaw at RT until condensation had evaporated. Slides were fixed in acetone for 10 s and then incubated in Gill’s haematoxylin for 2 min. Slide was then washed in running tap water for 1 min. Sections were incubated in Scott’s water for 1 min and subsequently washed again in tap water for 1 min. Afterwards, the sections were incubated with eosin for 45 s and then washed in tap water for a further 1 min. Sections were dehydrated first in 70% ethanol for 1 min, and then in 100% ethanol for 1 min, and finally in HistoClear (National Diagnostics) for an additional 1 min. Slides were mounted using DPX mounting medium.

### Electron microscopy

Mouse *soleus* muscle was obtained from five Zak^−/−^ and three controls. Each *soleus* was fixed individually for 20 min in fixative 2% glutaraldehyde, 2% formaldehyde in buffer (0.1 M sodium phosphate buffer pH 7.4) followed by further dissection cross sectionally into smaller blocks. All blocks were processed together by the Imaging and Cytometry team within the University of York Technology Facility as follows. Muscle samples were fixed for a further 1 h at ambient temperature in 2% glutaraldehyde, 2% formaldehyde fixative and then washed with buffer (3x 15 min) before secondary fixation in 1% osmium tetroxide (in buffer, 1 h on ice). Samples were subsequently dehydrated through a graded ethanol series before infiltration and embedding in Spurr resin (Taab laboratories, S0-24D). Sections (~70 nm) were collected on 200 mesh copper TEM grids and post stained with uranyl acetate (2% (w/v) in 50% ethanol) and lead citrate (48) before viewing using an FEI Tecnai 12 G2 fitted with a CCD camera.

### Scratch wound assay

WT and ZAK KO C2C12 cell lines (KOD9 and KOC10) were grown to 100% confluency in a six-well plate, three replicates per cell line. A 200 μl pipette tip was used to scrape a single vertical line through the cells from the growth surface (0 h). Cells were imaged every 3 h for a total of 24 h using an Evos XL core microscope (Thermo Fisher, AMEX1000). For quantification of wound closure, three images per time point were taken and averaged. To quantify wound closure, the space void of cells was calculated and taken away from the initial wound measurement at 0 h. An average of the distance travelled at each time point was then calculated compared with the initial wound distance at 0 h. A replica of this experiment was carried out using live cell imaging (Livecyte, Phasefocus), using the manufacturer’s automated software to generate images with a 10× objective for 30 h at 1-h intervals from cells maintained in an environmental chamber at 37°C with 5% CO_2_ and 95% humidity. Single-cell tracking quantifications were generated using the Livecyte integrated analysis suite.

### Phosphoproteomics

#### Sample preparation for MS analysis

A phosphoproteomic assay (adapted from (49)) was used to identify the immediate downstream targets of ZAKβ, whereby recombinant ZAKβ was added to a kinase-dead skeletal muscle lysate and subsequent phosphopeptides detected by LC–MS/MS.

TA muscle from WT C57Bl/6 mice was collected and snap-frozen in liquid nitrogen–cooled isopentane. Protein was extracted using the protocol as stated above. Skeletal muscle lysate was kept on ice and used fresh for the remainder of processing. Muscle extracts were treated with either FSBA (Sigma; F9128, 5 mm) dissolved in dimethyl sulphoxide (DMSO) or DMSO as control, to irreversibly inhibit all endogenous kinases. Skeletal muscle lysate treated with FSBA was desalted using Millipore Amicon ultrafiltration columns with a 3 kDa molecular weight cutoff (Merck; UFC500324) to remove all unreacted FSBA. When using the ultrafiltration columns, samples were diluted 1:10 with Nonidet P-40 buffer (10 mm Tris HCl, 10 mm NaCl, 3 mm MgCl_2_, 0.5% Nonidet P-40). Following desalting, samples diluted 1:5 with 5x kinase assay buffer (50 mm Tris (pH 7.2), 125 mm β-glycerophosphate, 250 mm KOH, 10 mm EGTA, 1.25 mm Na_3_VO_4_, 50 mm MgCl_2_ and 2 mm DTT, in ddH_2_O) and assayed for 3 h along with recombinant active ZAKβ (5 μg; obtained from Drs Hilary McLauchlan and James Hastie at the MRC PPU, University of Dundee) and ATP (Sigma, A26209) or [γ-32P]ATP (10 μCi; Perkin Elmer, NEG002H250UC).

For 1D SDS-PAGE analysis of skeletal muscle lysate treated with FSBA, [γ-32P] ATP and recombinant ZAKβ protein, the reactions were stopped using 4x NuPAGE LDS buffer and 10x NuPAGE Reducing Agent. Samples were electrophoresed using a 10% polyacrylamide gel at 200 V for 1 h or until the dye had run off the gel. Protein loading assessed using SafeBlue (NBS Biologicals, NBS-SB1L). X-ray film (Insight Biotechnology, sc-201 696) was placed over the gel encased in acetate and exposed for 5 days before being developed.

#### LC–MS/MS analysis

For LC–MS/MS, proteins were precipitated using 4x ice-cold acetone and left at −20°C overnight. Samples were then pelleted for 10 min at 15 000*g*. Once the supernatant was removed, the pellet was left to air-dry. The pellet was resuspended in urea lysis buffer (100 mm Tris (pH 7.4), 8 M Urea, 1 mm Na_3_VO_4_, 2.5 mm Na_4_P_2_O_7_ and 1 mm β-glycerophosphate) using sonication. 0.0364x the sample volume of DTT reducing solution (19.25 mg/ml DTT in ultra-pure water) was added to the samples and incubated at 55°C for 30 min. The samples were then cooled to 4°C for 10 min. 0.1x the sample volume of IAM alkylating solution (19 mg/ml iodoacetamide in ultra-pure water) was added and incubated in the dark at RT for 15 min. Samples were then diluted 4x with 50 mm ammonium bicarbonate and 1:50 (protease:protein m:m) protease (Trypsin and Lys-C mix). Samples were incubated ON at 37°C.

For peptide desalting, ON samples were acidified in 0.1% TFA. C18-E cartridges (Phonomenex) were loaded with 100% acetonitrile. The cartridge was then conditioned with 2 ml of 80% acetonitrile/0.1% TFA. Equilibration of the cartridge was performed using 2 ml of 0.1% TFA. Samples were loaded onto the cartridge using moderate pressure and flow rate. Column was washed with 2× 0.25 ml of 0.1% TFA. Phosphopeptides were eluted with 2× 0.2 ml of 80% acetonitrile/0.1% TFA and collected into an Eppendorf tube. Samples were subsequently dried using SpeedVac using moderate heat.

LC–MS/MS was performed over a 60-min acquisition with elution from a 50 cm EasyNano PepMap column onto a Fusion Orbitrap Fusion Tribrid mass spectrometer using a Waters mClass nanoUPLC. LC–MS chromatograms were imported into PEAKSX-Studio for peak picking and peptide identification. Data were searched against the mouse subset of the UniProt database. These data are filtered to 1% false discovery rate for identification. Complete mass spectrometry data sets and proteomic identifications are available to download from MassIVE (MSV000090935) (doi:10.25345/C5TB0Z08V) and ProteomeXchange (PXD037832).

### Raw data processing

The full complement of phosphopeptides identified in the FSBA phosphoproteomics screen were a combination of significant phosphopeptides with high probability of confidence being present in all five biological replicates and phosphopeptides identified in fewer than five replicates with reduced confidence. The complete data was processed in R (v4.0.3), to produce a list of significant phosphopeptides. Generation of the list of significant phosphopeptides was necessary to identify the potential targets of ZAKβ for further processing. The phosphoproteomic assay alone yielded a list of 114 S/T phosphopeptides from 48 individual proteins.

### *In silico* prediction of ZAKβ substrates

The phosphosite sequences obtained from our phosphoproteomic screen were used to predict further potential substrates of ZAKβ. A ZAKβ PSSM was generated by submitting an alignment of the 114 15-mer phosphopeptide sequences to the PSSMSearch website (http://slim.icr.ac.uk/pssmsearch/) using the default settings. The PSSM was then cross-referenced against the entire mouse proteome within the PSSMSearch website to identify further putative ZAKβ substrates. From this analysis, 1449 phosphopeptides from 1200 different proteins were identified. The resultant list was cross-referenced against a mouse skeletal muscle proteome for KEGG analyses using GProfiler (https://biit.cs.ut.ee/gprofiler/gost) or interactome analysis using STRING (https://string-db.org/). The list of putative ZAKβ targets from a site-specific peptide array was obtained from Johnson *et al*. (27). The raw data were filtered to include proteins within the top-ranking categories for ZAKβ activity. Disease enrichment and interaction analyses were performed using STRING.

#### Generation of fish line

Guide strand RNA targeting *Zakβ* in zebrafish were designed using ‘CHOPCHOP’ (https://chopchop.cbu.uib.no/) from exon 2 of the *Zakβ* gene. Two gene-specific 20–22 nt sequences were selected, each including a protospacer adjacent motif: (i) GCAGCTAATACGACTCACTATA**GGA**AAGGGATCTGAACGAAACGTTTTAGAGCTAGAAATA; (ii) GCAGCTAATACGACTCACTATA**GGT**CCCACAGGATAAAGAAGGTTTTAGAGCTAGAAATA. In addition to the target sequence, a T7 promoter region is incorporated into these forward primers. Templates are generated by PCR using the gene-specific and common reverse primers with the high-fidelity Phusion polymerase (Thermo Fisher), and guide strand RNA is synthesized using MEGA-shortscript T7 transcription kit (Ambion). 300 pg of the two gene-specific sgRNA together with 1 ng Cas9 protein in a 1 nl volume was injected into zebrafish embryos (AB WT) at the 1–4 cell stage. F0 zebrafish were raised to sexual maturity and outcrossed with WT AB zebrafish, F1s were genotyped by PCR of the relevant genomic DNA region and heterozygotes identified by heteroduplex analysis, and the specific disruption was identified by sequencing after cloning the PCR product into a plasmid. These F1s were backcrossed to AB before selecting F2 heterozygotes which were outcrossed again before raising a line of heterozygotes with a known mutation. Homozygous *Zakβ* zebrafish were generated by in-crossing heterozygotes and genotyping the offspring by fin-clipping at maturity.

### Methodology for swimming analysis

Comparison of key swimming attributes between *Zakβ^−/−^* zebrafish and siblings. *Zakβ^+/−^* and *Zakβ^−/−^* fish were raised in the same tank. The zebrafish were transferred to a recording tank, left to acclimatize for 10 min, before a 5-min video recording. A 30-s recording from the first half and a 30-s recording from the second half were processed using ShadowFish, and the tracked frames coordinates were processed for key swimming attributes using MATLAB: (a) distance travelled (cm); (b) velocity (cm/s); (c) percentage of time moving; (d) mean length of time for each swimming episode (s); (e) mean tail beat frequency (hertz); (f–h) mean tail beat frequency at low, medium and high speeds, respectively; (i) mean tail bend amplitude; and (j–l) the mean tail bend amplitude at low, medium and high speeds, respectively. Each data point is an individual recording; the bar chart and error bars are mean ± standard error of the mean. Twelve WT and eight *Zakβ^+/−^* zebrafish had recordings taken, providing 24 data points for WT and 16 data points for *Zakβ^+/−^*. In the case of 18-month-old fish, a ROUT test was performed to eliminate anomalies. Six WT and eight *Zakβ^−/−^* zebrafish recordings were taken, providing 12 data points for WT and 16 data points for *Zakβ^−/−^* zebrafish. The ROUT test for anomalies eliminated two data points for WT for distance travelled and two data points for mean velocity. For 35-month-old fish, recordings were taken from four WT and five *Zakβ^−/−^* zebrafish that had been kept in separate tanks, providing 8 data points for WT and 10 data points for *Zakβ^−/−^* zebrafish.

### Mice

Generation of *Zak^−/−^* mouse line as described in Nordgaard *et al*. (13). Mice were housed in individually ventilated cages at the animal facility at the University of York. Research was approved and monitored by the UK Home Office. All animal regulated procedures were carried out according to Project License constraints (PEF3478B3) and Home Office guidelines and regulations. Mice were also housed at the animal facility of the Department of Experimental Medicine at the University of Copenhagen, and the research was monitored by the Institutional Animal Care and Use Committee. All of the mouse work was performed in compliance with Danish and European regulations. Animal experiments were approved by the Danish Experimental Animal Inspectorate. Mice were kept on a 12-h light/12-h dark cycle in ventilated cages at RT and fed regular rodent chow.

### Patient

The patient originated from France, and sample collection was performed with written informed consent from the patient and participating family members, according to the Declaration of Helsinki.

### Statistics

To measure the effect of loss of function of ZAK on *soleus* and TA muscle regeneration *in vivo*, 10× magnification images were captured using a Leica brightfield microscope. The number of fibres displaying centralized nuclei was expressed as a percentage of the total number myofibres to create one single data point per image. Comparison of percentage of centralized nuclei determined from full coverage of *soleus* (*n =* 3) and TA muscles (*n =* 6). An average of the percentage of fibres with centralized nuclei was calculated across three mice per condition. *Soleus* muscle fibre size determined from complete coverage of *soleus* muscle (*n =* 3). TA fibre size determined from a minimum sample of 2000 fibres from sections taken at the same level (*n =* 6). Data were compared using either the Student’s *t*-test or one-way ANOVA test followed by a Bonferroni HSD *post hoc* test. *P*-values < 0.05 were deemed significant.

For fibre typing of *soleus* muscle sections, relative numbers of type I and type IIa fibres were determined manually by counting the total of fibres expressing each myosin isoform.

The fibre cross-sectional area was calculated as an average of three individual muscles. To avoid regional fibre size variation in the TA muscle to confound comparisons, a scale factor (average area of electroporated: average area non-electroporated) was obtained from at least three electroporated regions within the muscle, which were then averaged to produce a single data point per muscle.

For morphological analyses of H&E-stained sections, 10× magnification images were captured using the Leica brightfield microscope (DM2500), camera and imaging software (SPOT Insight FireWire; Diagnostic instruments). Centralized nuclei were counted manually. The number of fibres displaying centralized nuclei was expressed as a percentage of the total number of myofibres to create one single data point per muscle. An average of the percentage of fibres with centralized nuclei was calculated across three mice per genotype. Data were compared using a one-way ANOVA test followed by a Tukey’s HSD *post hoc* test. *P*-values < 0.05 were deemed significant.

To measure the effect of loss of function of ZAK on TA muscle regeneration *in vivo* in response to BaCl_2_-induced injury, 10× magnification images were captured using a Leica brightfield microscope from 28 dpi muscles. The fibres displaying centralized nuclei were used as a proxy for injured muscle fibres undergoing regeneration (50). Twenty-eight days post-injury TA fibre size was determined from a measurement of all centronucleated fibres (minimum of 750 fibres per section) from sections taken at the same level (*n =* 4). Data were compared using Student’s *t*-test. A *P*-value < 0.05 was deemed significant.

*Conflict of interest statement*. None declared.

## Supplementary Material

Supp_Fig_1A_B_ddad113

Supp_Fig_2A_B_ddad113

Supp_Fig_3_ddad113

Supp_Fig_4_ddad113

Supp_Fig_5_ddad113

Supp_FIg_6_ddad113

Supp_Fig_7_ddad113

Supp_Fig_8_ddad113

Supp_Fig_9_ddad113

Supp_Fig_10_ddad113

Supp_table_1_Direct_ZAK_targets_ddad113

Supp_table_2_Predicted_targets_of_ZAKb_ddad113

Supp_table_3_KEGG_analysis_of_extended_ZAKb_targets_ddad113

Supp_table_4_Disease_associated_proteins_ranked_as_number_1_ZAK_targets_ddad113

Supp_table_5_Disease_associated_proteins_in_222_protein_overlap_ddad113

Supp_table_6_KEGG_analysis_of_TA_DEGs_ddad113

Supp_table_7_KEGG_analysis_of_Soleus_DEGs_ddad113

Supplementary_Figure_Legends_ddad113

## Data Availability

The data that support the findings of this study are available upon reasonable request.
