# Correction to: Myofibrillar myopathy hallmarks associated with ZAK deficiency

**DOI:** 10.1093/hmg/ddad168

**Published:** 2023-10-03

This is a correction to:

Amy Stonadge, Aitana V Genzor, Alex Russell, Mohamed F Hamed, Norma Romero, Gareth Evans, Mary Elizabeth Pownall, Simon Bekker-Jensen, Gonzalo Blanco, Myofibrillar myopathy hallmarks associated with ZAK deficiency, *Human Molecular Genetics*, Volume 32, Issue 17, 1 September 2023, Pages 2751–2770, https://doi.org/10.1093/hmg/ddad113

In the originally published version of this manuscript, author, Mary Elizabeth Pownall, was denoted incorrectly.

This error has been corrected.

