# Supplementary figures and images for: Myofibrillar myopathy hallmarks associated with ZAK deficiency
Source: Hum Mol Genet. 2023 Jul 10;32(17):2751–70. doi: 10.1093/hmg/ddad113 (PMC10789240; doi:10.1093/hmg/ddad113)

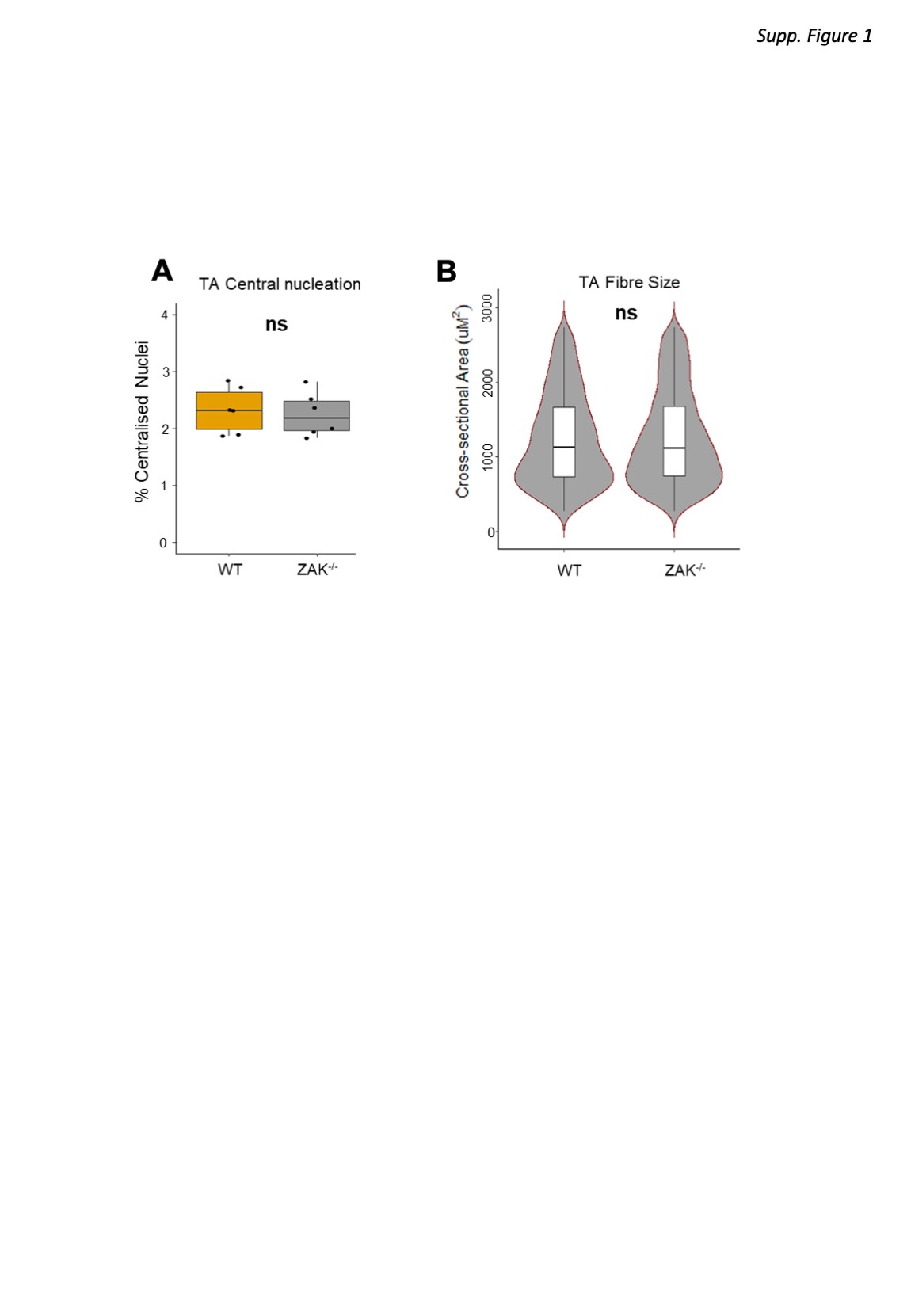

Supplement: Supp_Fig_1A_B_ddad113 [file supp_fig_1a_b_ddad113.jpeg]

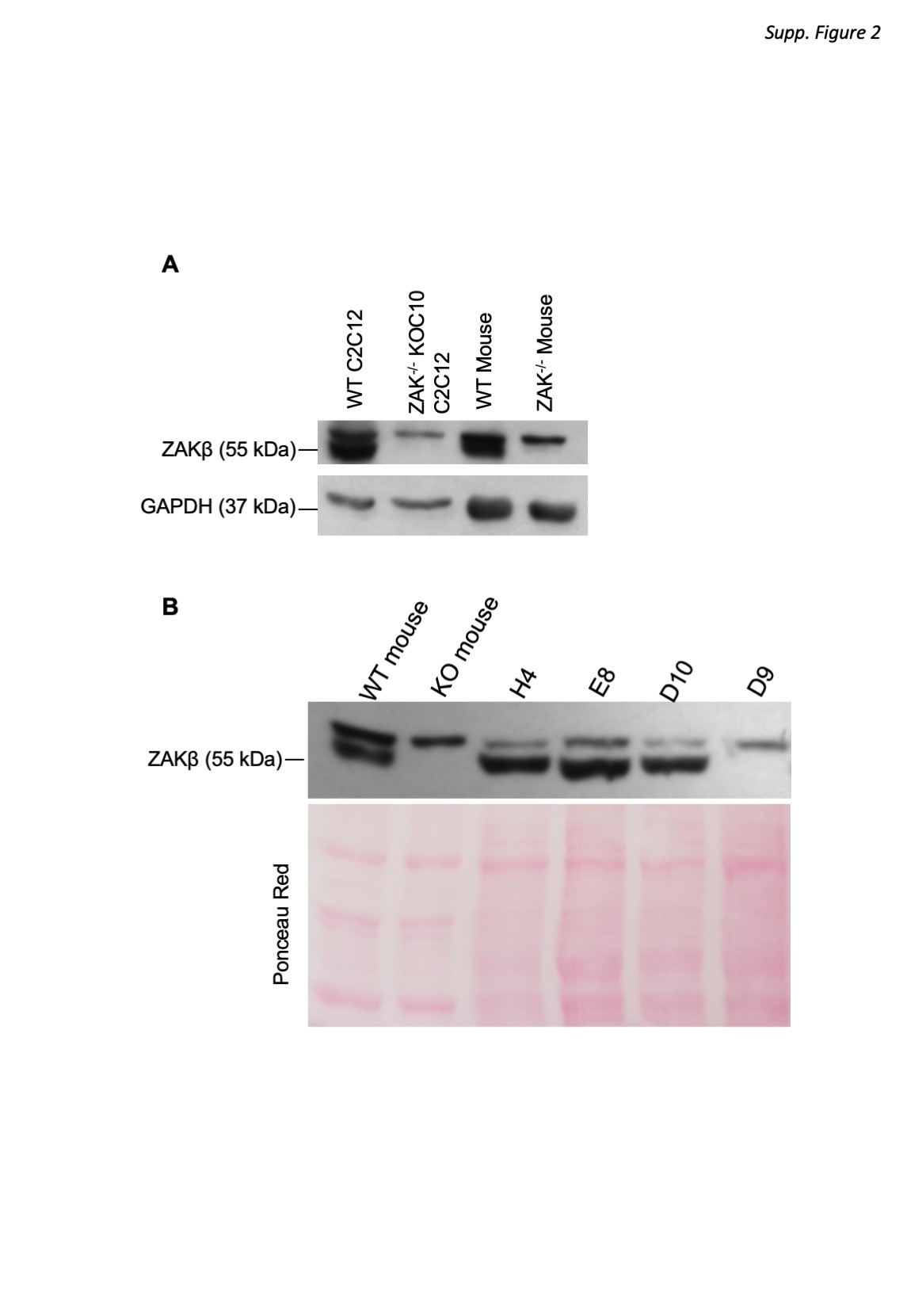

Supplement: Supp_Fig_2A_B_ddad113 [file supp_fig_2a_b_ddad113.jpeg]

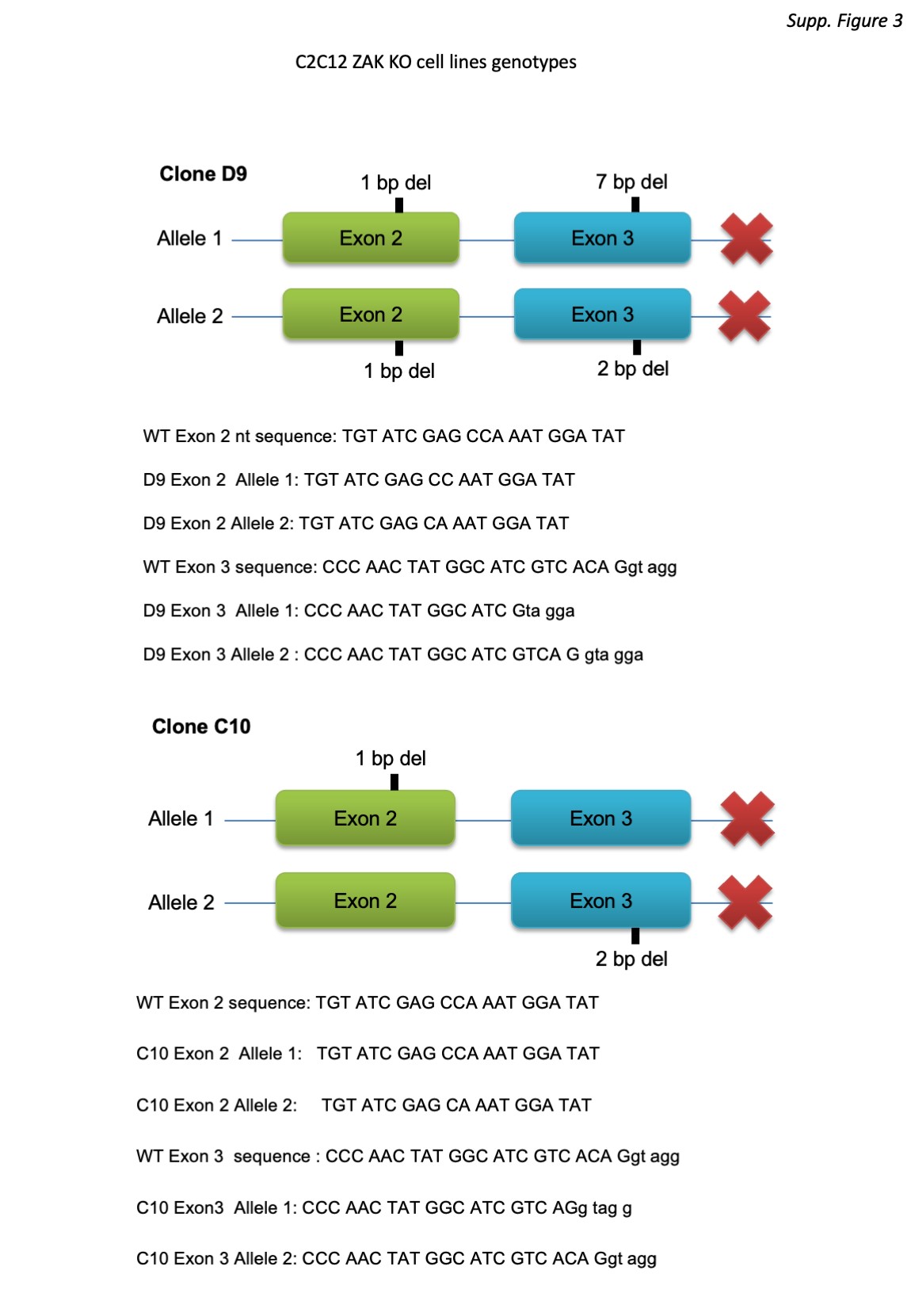

Supplement: Supp_Fig_3_ddad113 [file supp_fig_3_ddad113.jpeg]

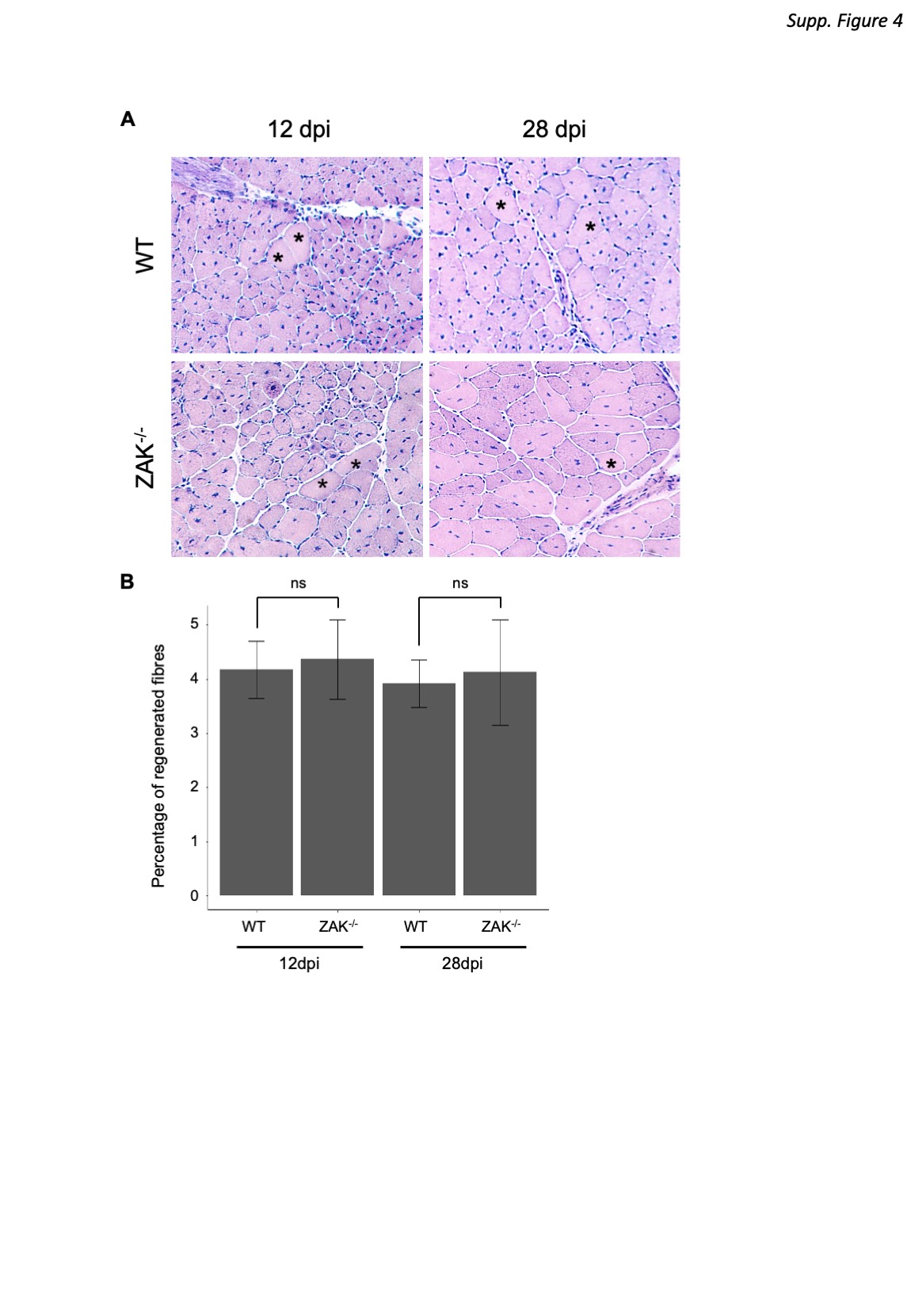

Supplement: Supp_Fig_4_ddad113 [file supp_fig_4_ddad113.jpeg]

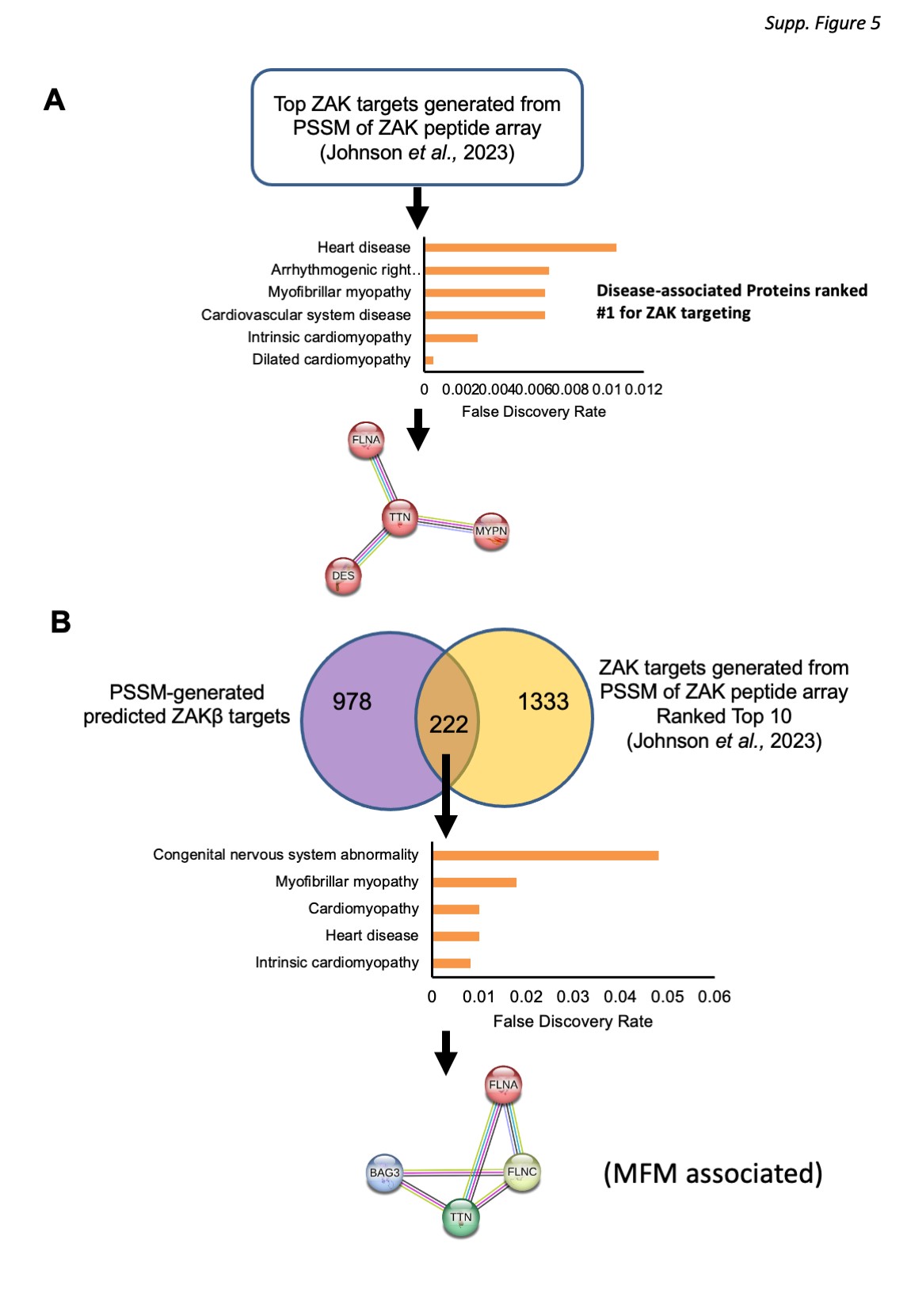

Supplement: Supp_Fig_5_ddad113 [file supp_fig_5_ddad113.jpeg]

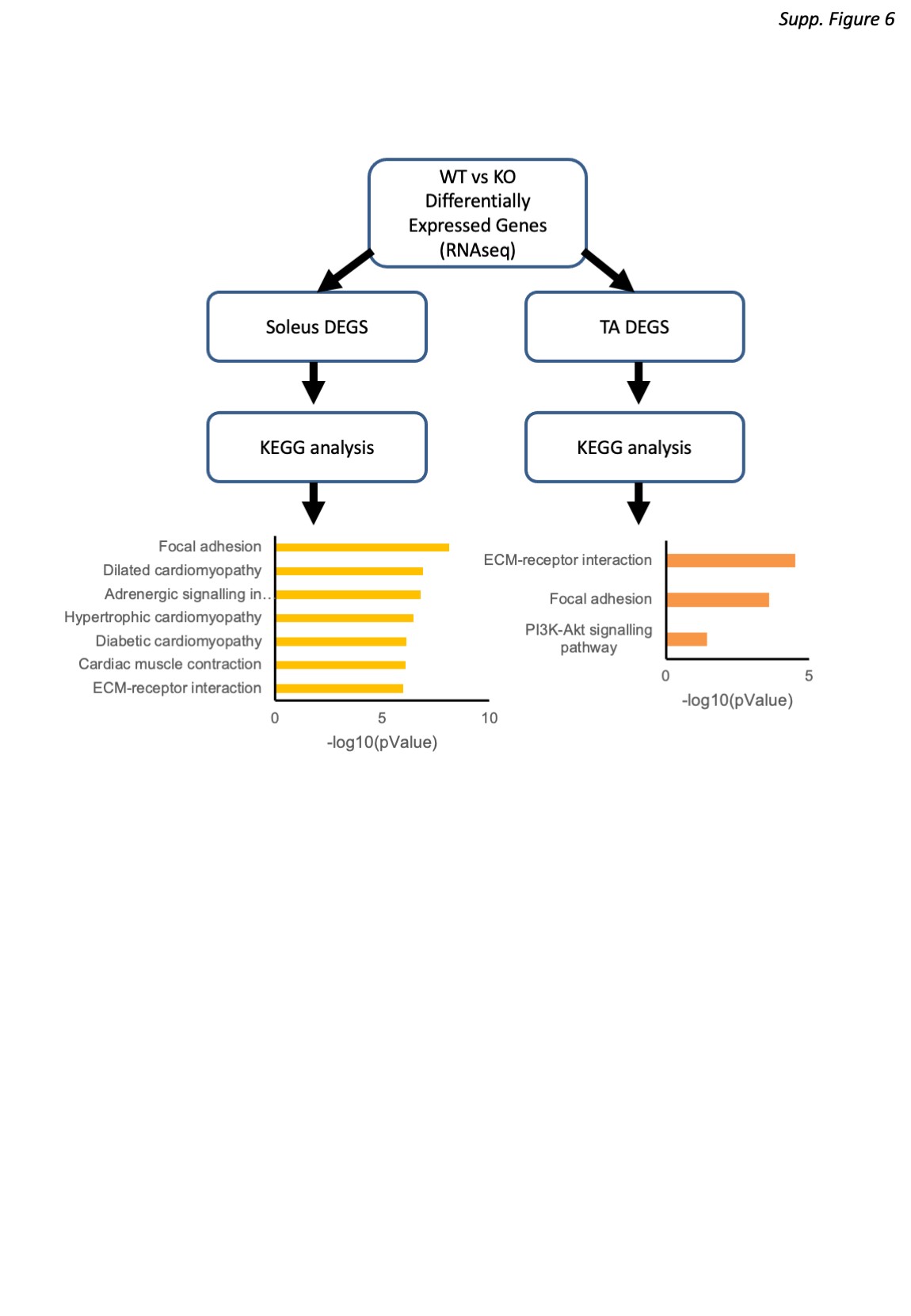

Supplement: Supp_FIg_6_ddad113 [file supp_fig_6_ddad113.jpeg]

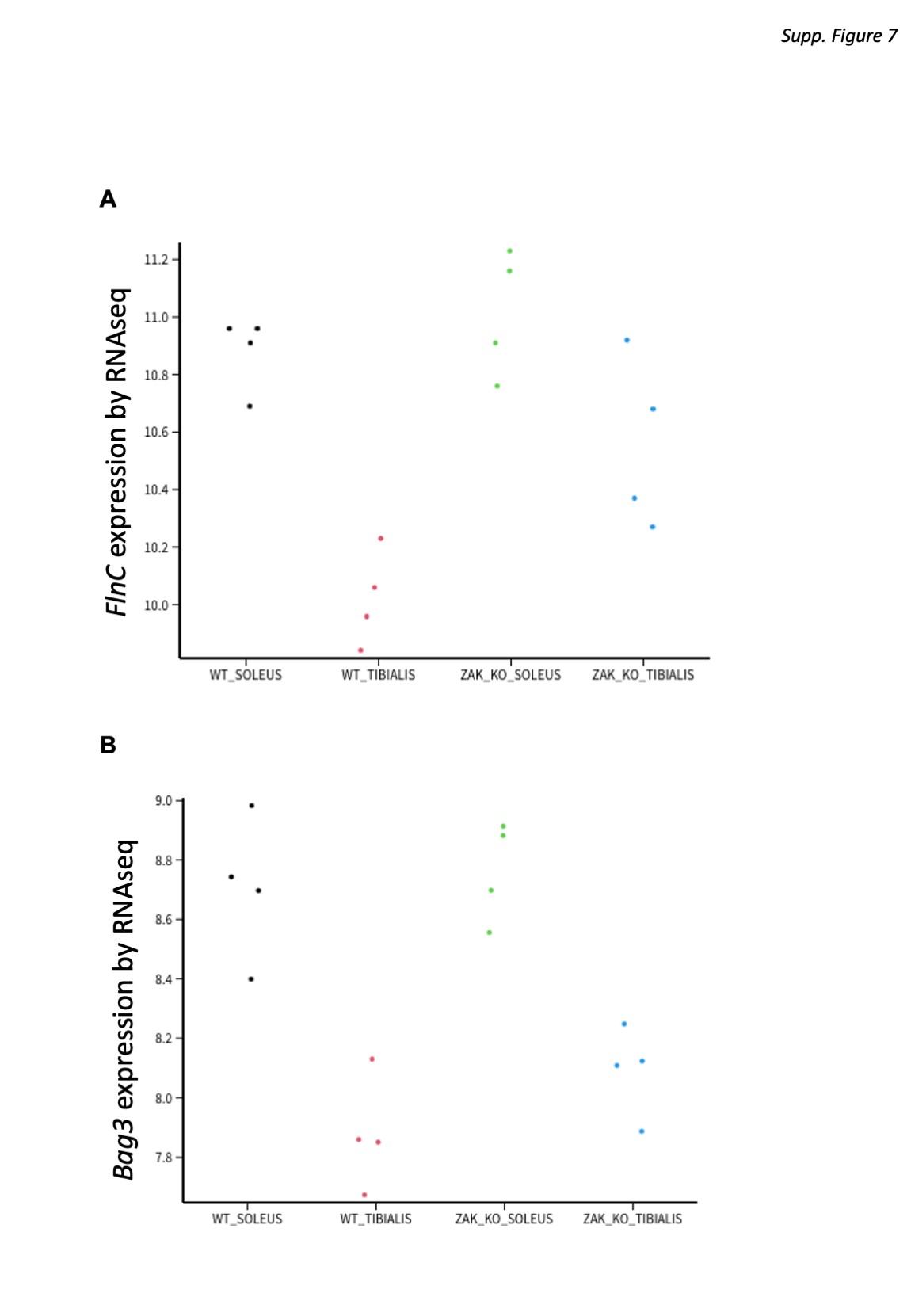

Supplement: Supp_Fig_7_ddad113 [file supp_fig_7_ddad113.jpeg]

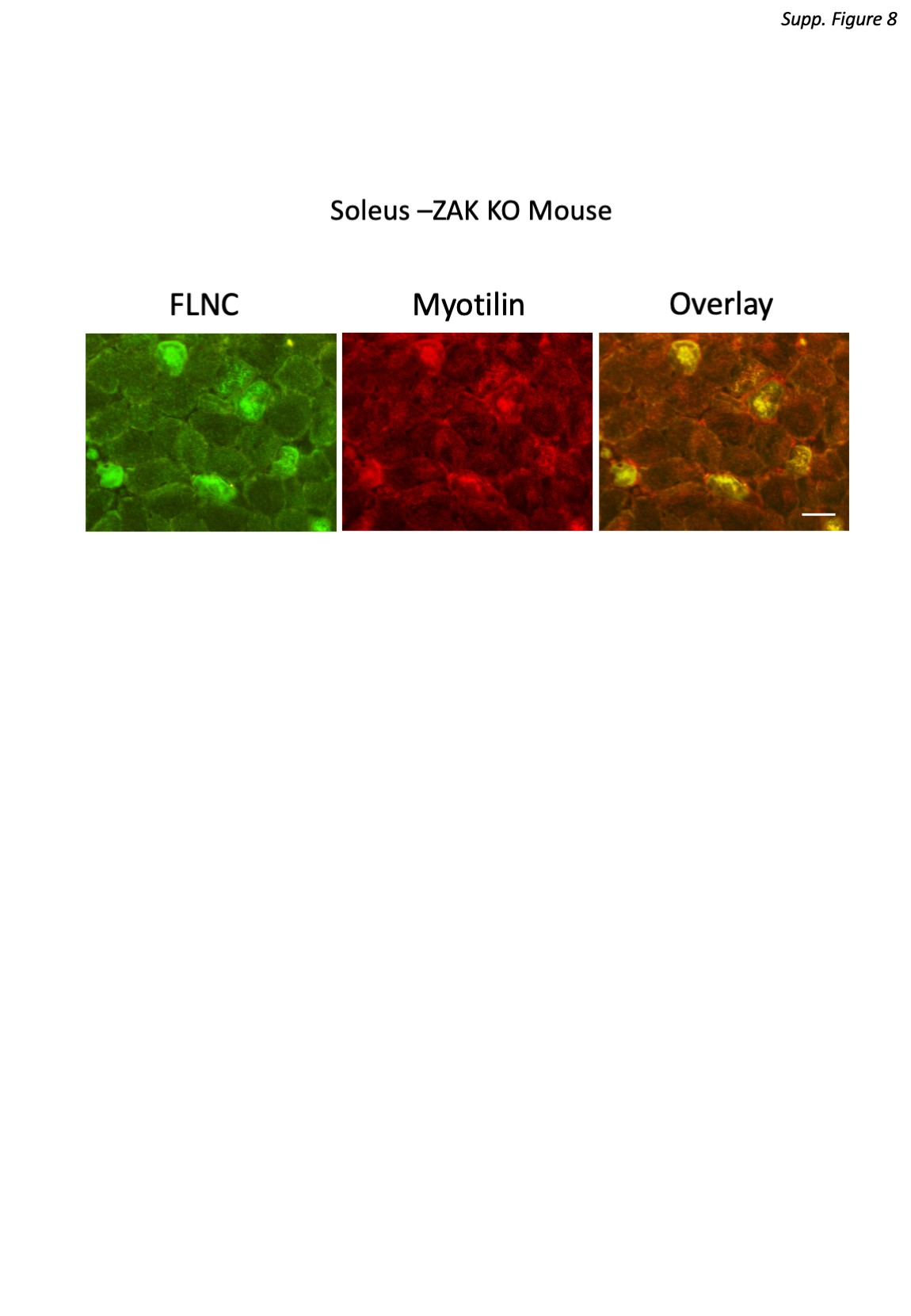

Supplement: Supp_Fig_8_ddad113 [file supp_fig_8_ddad113.jpeg]

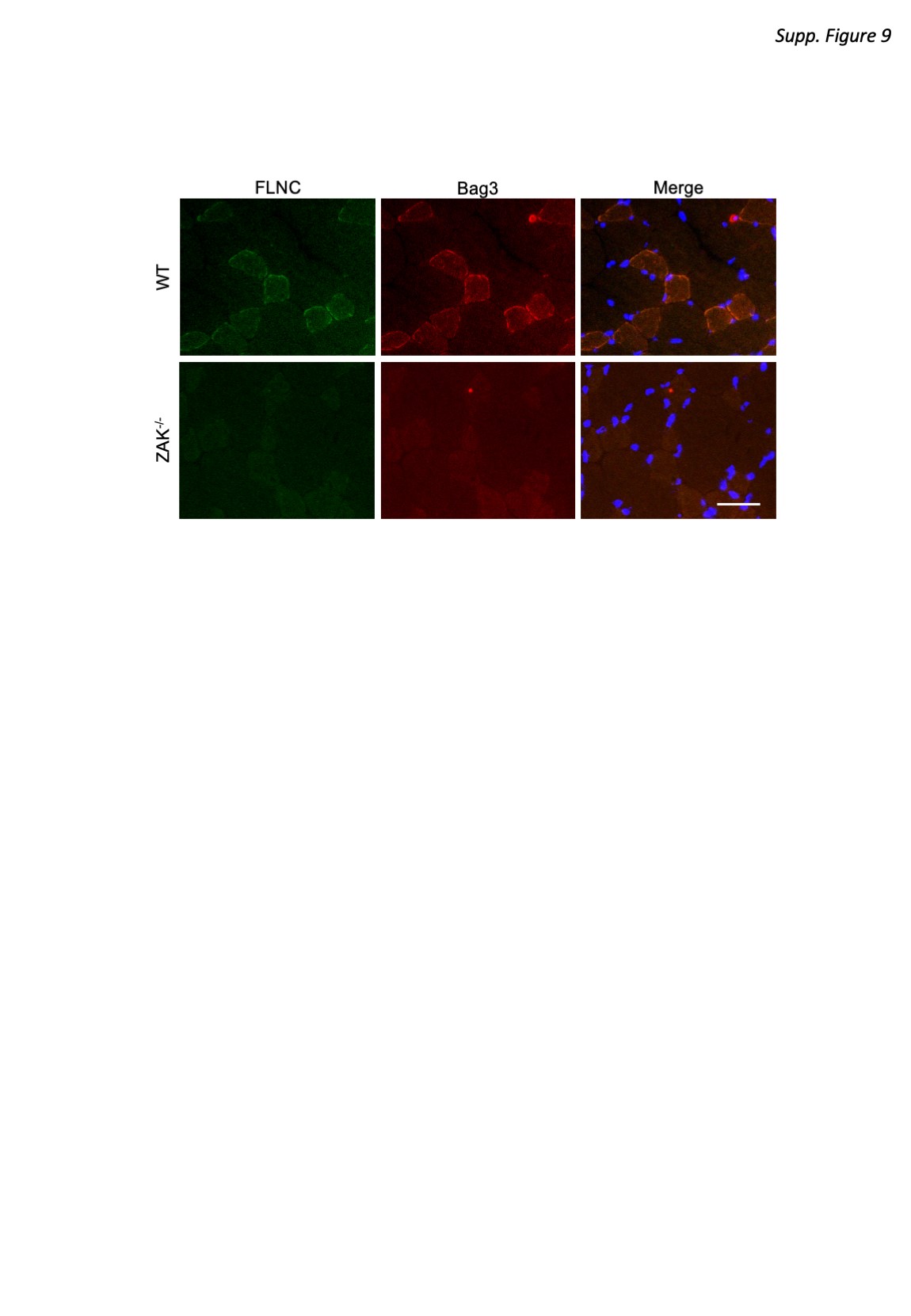

Supplement: Supp_Fig_9_ddad113 [file supp_fig_9_ddad113.jpeg]

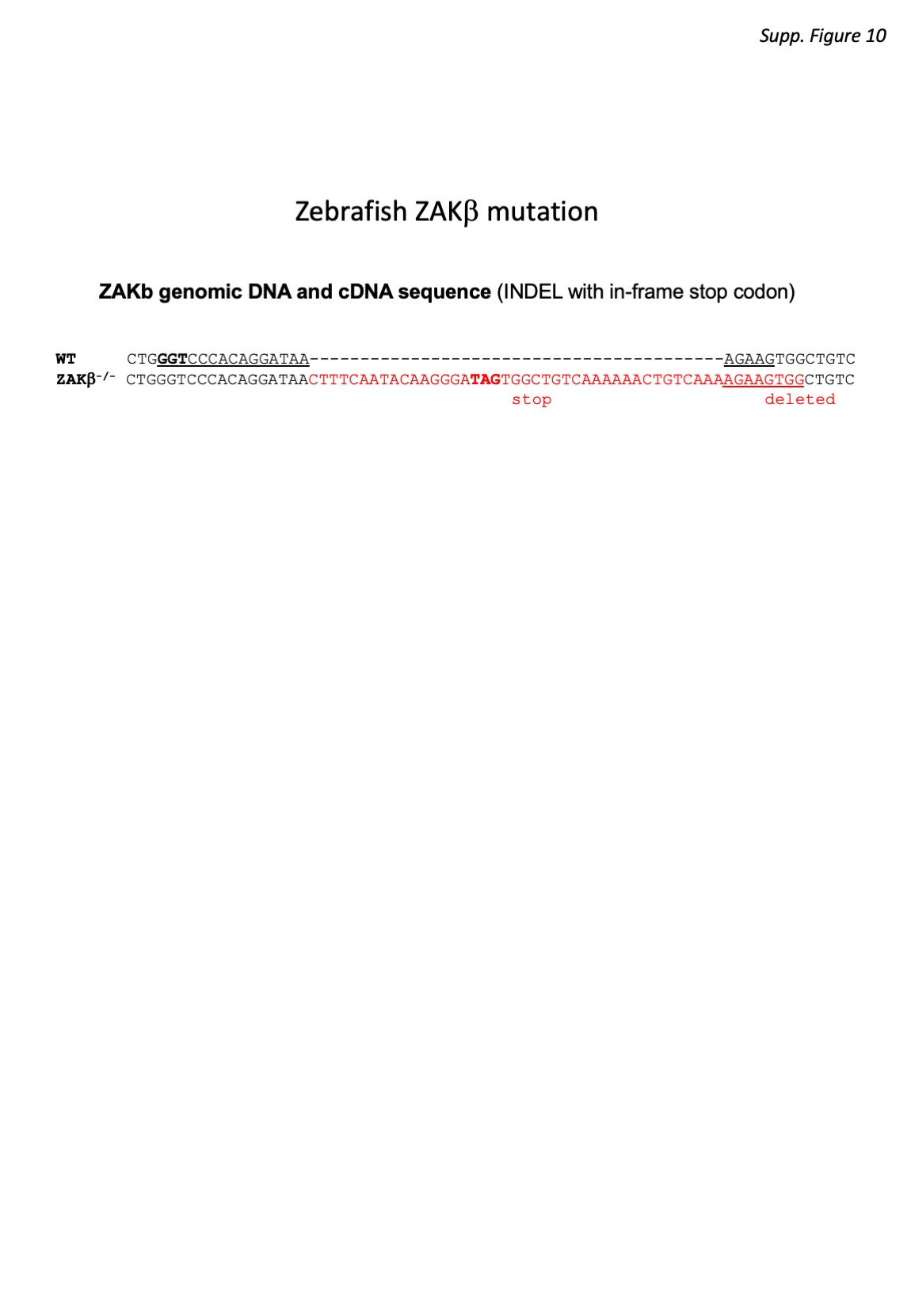

Supplement: Supp_Fig_10_ddad113 [file supp_fig_10_ddad113.jpeg]
