# Supplementary material for: Myofibrillar myopathy hallmarks associated with ZAK deficiency
Source: Hum Mol Genet. 2023 Jul 10;32(17):2751–70. doi: 10.1093/hmg/ddad113 (PMC10789240; doi:10.1093/hmg/ddad113)
Supplement: Supplementary_Figure_Legends_ddad113 [file supplementary_figure_legends_ddad113.docx]

**Supplementary Figure 1.** Neither fibre atrophy or central nucleation was identified in the *tibialis anterior* (TA) muscle from 8-week-old *Zak^-/-^* or control mice. (A) Box plots showing quantifications of fibres with centralised nuclei in *Zak^-/-^* and control mice (n=6, individual data points shown). Independent t-test, (*) p < 0.001. (B) Violin plots showing quantifications of fibre cross-sectional area from H&E staining of TA muscle cross sections from 8-week-old *ZAK^-/-^* and control mice (n = 5). Independent t-test, (*) p < 0.001.

**Supplementary Figure 2: Generation of CRISPR-Cas9-mediated ZAK knockout C2C12 cell line.** (A) Protein extracts were obtained from WT and ZAK KO (KOC10) cell lines 14 days post differentiation and run alongside validated protein extracts from WT and *Zak^-/-^* mouse *gastrocnemius/soleus* muscles on SDS-PAGE. ZAKβ is not detected in the KOC10 clone. Protein loading assessed using GAPDH. (B) Protein extracts were obtained from WT and ZAK KO (KOD9) cell lines 14 days post differentiation and run alongside validated protein extracts from WT and *Zak*^-/-^ mouse *gastrocnemius/soleus* muscles on SDS-PAGE. *Zakβ* is not detected in the KOD9 clone. Protein loading assessed using Ponceau Red staining.

**Supplementary Figure 3:** Mutations identified in clones D9 and C10 within the *ZAK* gene exons 2 and 3, as indicated.

**Supplementary Figure 4:** (A) Representative H&E images of regenerating muscle 12 and 28 days post barium chloride injury (dpi). Asterisks depict fully regenerated fibres which are not centrally nucleated surrounded by regenerating fibres identified by centralised nuclei. (B) A histogram showing the percentage of fully regenerated fibres within the regenerating area of barium chloride-induced acute muscle injury at both 12 and 28 days post injury. Student’s t-test; (ns) = non significant.

**Supplementary Figure 5: PSSM generated by peptide array identifies myofibrillar myopathy as disease associated with ZAK targets.** (A) The ZAK PSSM generated in (31) identified proteins associated with myofibrillar myopathy as highly enriched in direct ZAK targets. (B) STRING analysis of the overlap between the proteins identified in the extended list in this study (1200 proteins) and the top 10 for direct ZAK targets identified in (31) (1555 proteins) revealed an enrichment of disease proteins associated with myofibrillar myopathy.

**Supplementary Figure 6**. Analysis of differentially expressed genes (DEGs) in the *soleus* and TA muscles from *Zak^-/-^* and control mice, from previously deposited RNAseq data (Nordgaard et al., 2022). KEGG analysis shows Focal adhesion and ECM-receptor interactions are processes significantly enriched in the phosphoproteomics and RNAseq lists (full lists of contributing genes in supplementary tables 4 and 5).

**Supplementary Figure 7: *Zak^-/-^* mouse skeletal muscle shows no difference in *Flnc* and *Bag3* transcript levels.** Levels of *Flnc* and *Bag3* were evaluated using RNAseq of both WT and *Zak^-/-^* mouse *soleus* and *tibialis anterior muscle*. (A) *Flnc* transcript levels do not change when ZAK is lost. (B) *Bag3* transcript levels are consistent in the WT and the *Zak^-/-^* mouse. Data extracted from PRJNA816072 (Nordgard et al, 2022).

**Supplementary Figure 8: Loss of ZAK causes FLNC and Myotilin rich accumulations in 8-week-old *soleus* muscle.** Representative immunofluorescence images of *soleus* muscle cross sections of 8-week-old WT and *Zak^-/-^* female mice stained with anti-FLNC and anti-MYOT. FLNC and Myotilin display highly reactive immunostaining in the same fibres. Scale bar represents 100 μm.

**Supplementary Figure 9: Loss of ZAK does not cause FLNC or BAG3 positive fibres in 8-week-old TA muscle.** Representative immunofluorescence images of TA muscle cross sections of 8-week-old WT and *Zak^-/-^* female mice stained with anti-FLNC and anti-BAG3. Scale bar represents 100 μm.

**Supplementary Figure 10: Description of gene targeting ZAKb^-/-^ in zebrafish.** *Danio rerio* ZAKβ gene, located on Chromosome 6, (ENSDARG00000044615.8), has 12 exons with the start codon in exon 2. Guide strand RNA was synthesized and co-injected with Cas9 protein into early embryos to target exon 2 upstream of the kinase domain. After raising embryos to maturity, breeding and screening the resulting fish using heteroduplex analysis (Zhu et al, 2014) a line of zebrafish carrying a disruptive mutation in ZAKβ was identified. The sequence of the mutated gene and cDNA sequence from mRNA extracted from mutant embryos and mutant adult muscle tissue is shown. The red text indicated the INDEL, where the inserted region includes an in-frame stop codon and the underlined red text indicates nucleotides that have been deleted.
